# Multigenetic Pharmacogenomics–Guided Treatment vs Treatment As Usual Among Hospitalized Men With Schizophrenia: A Randomized Clinical Trial

**DOI:** 10.1001/jamanetworkopen.2023.35518

**Published:** 2023-10-06

**Authors:** Zhewei Kang, Ying Qin, Yutao Sun, Zhe Lu, Yaoyao Sun, Huan Chen, Xiaoyang Feng, Yuyanan Zhang, Hua Guo, Hao Yan, Weihua Yue

**Affiliations:** 1 Institute of Mental Health, Peking University Sixth Hospital, Beijing, China; 2 National Clinical Research Center for Mental Disorders, Peking University Sixth Hospital, Beijing, China; 3 NHC Key Laboratory of Mental Health, Research Unit of Diagnosis and Treatment of Mood Cognitive Disorder, Chinese Academy of Medical Sciences, Beijing, China; 4 The Second People’s Hospital of Guizhou Province, Guiyang, China; 5 Tangshan Mental Health Center, Tangshan Fifth Hospital, Tangshan, China; 6 Shantou University Mental Health Center, Shantou, China; 7 Zhumadian Second People’s Hospital, Zhumadian, China; 8 PKU-IDG/McGovern Institute for Brain Research, Peking University, Beijing, China; 9 Chinese Institute for Brain Research, Beijing, China

## Abstract

**Importance:**

Limited evidence supports multigenetic pharmacogenomics–guided treatment (MPGT) in schizophrenia.

**Objective:**

To evaluate the clinical effectiveness of MPGT in schizophrenia in a randomized clinical trial (RCT).

**Design, Setting, and Participants:**

This RCT was conducted from March 2020 to March 2022. Male Chinese Han inpatients aged 18 to 60 years diagnosed with schizophrenia with a Positive and Negative Symptom Scale (PANSS) score of 60 or more from 2 selected study hospitals were included. Patients and raters were masked to MPGT or treatment as usual (TAU) randomization.

**Interventions:**

Participants were randomly assigned in a 1:1 ratio to receive either MPGT or TAU for 12 weeks.

**Main Outcomes and Measures:**

The primary efficacy outcome was the percentage change in PANSS total scores (range, 30 to 210) from baseline to week 6 analyzed by a modified intention-to-treat mixed model for repeated measures. The secondary outcome included response and symptomatic remission rates.

**Results:**

A total of 210 participants (mean [SD] age, 29.2 [8.8] years) were enrolled and analyzed, with 113 assigned to MPGT and 97 to TAU. Compared with those randomized to TAU, participants randomized to MPGT demonstrated a significantly higher percentage change in PANSS score (74.2% vs 64.9%; adjusted mean difference, 9.2 percentage points; 95% CI, 4.4-14.1 percentage points; *P* < .001) and a higher response rate (93 of 113 [82.3%] vs 63 of 97 [64.9%]; adjusted odds ratio, 2.48; 95% CI, 1.28-4.80; *P* = .01) at the end of week 6.

**Conclusions and Relevance:**

In this RCT of MPGT, MPGT was more effective than TAU in treating patients with schizophrenia. These findings suggest that multigenetic pharmacogenomic testing could serve as an effective tool to guide the treatment of schizophrenia.

**Trial Registration:**

Chinese Clinical Trial Registry Identifier: ChiCTR2000029671

## Key Points

**Question:**

What is the therapeutic efficacy of multigenic pharmacogenomics–guided treatment in patients with schizophrenia?

**Findings:**

In this randomized clinical trial that included 210 Chinese Han men, patients treated with multigenetic pharmacogenomics–guided treatment had a greater symptom improvement than those treated with treatment as usual after a 6-week treatment, measured as the mean difference in percentage change of Positive and Negative Syndrome Scale score.

**Meaning:**

Multigenetic pharmacogenomic testing can be an effective tool to guide the treatment of schizophrenia.

## Teaser

This randomized clinical trial evaluates the clinical effectiveness of multigenetic pharmacogenomics–guided treatment in schizophrenia among Han men in China.

## Introduction

Schizophrenia is a complex polygenic disorder characterized by disturbances in multiple mental modalities.^1^ It affects approximately 24 million people worldwide,^2^ causing significant burdens on individuals, families, and society.^3^ In clinical practice, antipsychotic medication is the first-line treatment for schizophrenia.^4^ However, only around 20% to 50% of patients respond well to antipsychotics,^5,6,7^ while many medications have substantial adverse effects profiles.^8^ The trial-and-error approach is frequently necessary prior to finding effective treatment, leading to treatment delays that can potentially affect patient adherence and worsen the disease.^9^ Therefore, there is an urgent need for reliable predictors of antipsychotic medication response and adverse effects to guide individualized therapy.

In recent years, pharmacogenomic testing has been widely used to guide drug therapy and has yielded exciting results.^10,11^ Pharmacogenomics studies the effects of genes and genetic variation on how an individual responds to certain medications or combinations of medications.^11^ By detecting these variations, pharmacogenomic testing can provide valuable information for medication selection and dose adjustment in clinical practice. Several commercial multigenetic pharmacogenomic tests, such as GeneSight^12^ and Genecept,^13^ have been developed and implemented in the clinical practice of psychiatry. These tests detect a combination of several genes and provide reports on drug recommendations using proprietary algorithms,^14^ which were found to perform better than single-variant testing in terms of predicting outcomes.^15^ A few randomized clinical trials (RCTs) found that multigenetic pharmacogenomics–guided treatment (MPGT) can yield better outcomes for patients with major depressive disorder.^16,17,18^ However, studies have mainly focused on antidepressants^19^ and there is still a lack of evidence of the effect of MPGT on schizophrenia.

Based on these foundations, we conducted an RCT to evaluate the efficacy of MPGT in male Chinese Han patients diagnosed with schizophrenia compared with treatment as usual (TAU). We hypothesized that MPGT would lead to a better improvement of symptoms.

## Methods

### Study Design and Participants

This study is a 12-week, 2-center, parallel RCT evaluating the therapeutic effects of MPGT compared with TAU in patients with schizophrenia. Patients and raters were masked to MPGT or TAU randomization. The trial protocol can be found in Supplement 1.

Participants were enrolled at the Peking University Sixth Hospital, Beijing, China, and the Second People’s Hospital of Guizhou Province, Guiyang, China, between March 2020 and March 2022. All participants were male Chinese Han inpatients aged 18 to 60 years who met the *Diagnostic and Statistical Manual of Mental Disorders* (Fourth Edition)^20^ criteria for schizophrenia and had a minimum score of 60 on the Positive and Negative Syndrome Scale (PANSS).^21^ Participants were excluded if they had concurrent substance use disorders (except nicotine dependence), neurological conditions (epilepsy, major neurocognitive disorders), other comorbid axis I psychiatric diagnoses, or severe unstable organic illnesses (major cardiovascular pathologies, uncontrolled diabetes, liver failure, and kidney failure).

The protocols of this study were approved by the Medical Ethical Committee of the Sixth Hospital of Peking University and the Medical Ethical Committee of the Second People’s Hospital of Guizhou Province. Written informed consent from all participants or their guardians was obtained. The RCT conformed to the Consolidated Standards of Reporting Trials (CONSORT) reporting guideline.^22^

### Randomization and Masking

Participants enrolled were randomly assigned to the MPGT or the TAU group in a 1:1 ratio using a preplanned randomization list. Raters were masked to the study group. Participants were masked to the study group and their pharmacogenomics report until the end of the trial. Clinicians who took care of participants in the MPGT group had access to the pharmacogenomics report to guide medication selection.

### Multigenetic Pharmacogenomic Testing

In this study, we detected single-nucleotide variant loci of 26 alleles or variants across 11 genes that are reported to be associated with antipsychotic medication metabolism, efficacy, or adverse effects (eTable 1 in Supplement 2). Genomic DNA was isolated from buccal samples, and genotyping examinations were conducted by Conlight Medical Inc, using the MassArray (MALDI-TOF MS) genotyping method. We used an algorithm where different weight values were assigned to each variant (eTable 2 in Supplement 2) to evaluate the gene-drug interaction. The algorithm was based on licensed technologies disclosed in issued patents (eAppendix in Supplement 2). It categorizes the medications into 3 categories for each participant: (1) use as directed, indicating a minimal or no gene-drug interaction, allowing physicians to use the medication directly in appropriate circumstances; (2) moderate gene-drug interaction, suggesting the medication should be used after evaluation; and (3) significant gene-drug interaction, indicating that the medication should be used under blood concentration monitoring or consider alternations (eTable 3 in Supplement 2). Detailed gene selection, interpretation, and a comprehensive description of the algorithm are provided in the eMethod in Supplement 2. A total of 8 commonly used atypical antipsychotics (amisulpride, aripiprazole, clozapine, olanzapine, paliperidone, quetiapine, risperidone, and ziprasidone) were included in our study.

### Trial Procedure

Detailed procedures are described in the eMethods in Supplement 2. In brief, participants who met the criteria were randomized 1:1 into 2 groups (MPGT and TAU). All participants had the multigenetic pharmacogenomic testing and received a low-dose treatment (2 mg per day or less) of risperidone or an equivalent-dose conversion of other antipsychotic medication for 1 week. At the end of the first week (when the pharmacogenomics report was available), participants in the MPGT group received antipsychotic medications recommended by the report of pharmacogenomic testing, while participants in the TAU group received antipsychotic medications according to the experience of clinical physicians. The use of nonpsychiatric medications was not restricted, except for inhibitors of the CYP2D6 enzyme (eTable 4 in Supplement 2). Follow-up assessments were conducted at the end of week 2, 6, and 12 after treatment assignment.

### Outcome Measures

The primary efficacy outcome of this trial was the percentage PANSS score change from baseline to the end of week 6. Percentage PANSS score change was calculated using the difference between the baseline and follow-up scores divided by the baseline score minus 30.

The secondary outcome was response or remission rates at each time point. Early response was defined as a percentage PANSS score change of 20% or more at the end of week 2,^23^ response was defined as a percentage PANSS score change of 50% or more^24^ at the end of week 6, and symptomatic remission was defined as a PANSS score of 3 or less on items P1 (delusions), P2 (conceptual disorganization), P3 (hallucinatory behavior), N1 (blunted affect), N4 (passive/apathetic social withdrawal), N6 (lack of spontaneity and flow of conversation), G5 (mannerisms and posturing), and G9 (unusual thought content) at the end of week 12.^25^

There were several exploratory outcome measurements. Patients in the MPGT group were divided into subgroups of patients with first-episode schizophrenia and relapsed schizophrenia to compare their therapeutic efficacy. We also evaluate the distribution of pharmacogenomic recommendation levels in the TAU group and compared the differences in treatment efficacy among the recommendation levels. The dose of medications and metabolic profile, including triglyceride, cholesterol, high-density lipoprotein cholesterol, low-density lipoprotein cholesterol, fasting plasma glucose (FPG), and prolactin, were evaluated.

### Power Calculations

Based on a previous study of pharmacogenomics-guided treatment on major depressive disorder,^17^ a moderate effect size (Cohen *d*) of 0.41 was suggested. With 210 participants, the power was 0.90 for a Cohen *d* of 0.40 for 2-tailed α less than .05 in examining hypothesized differences between MPGT and TAU. The power calculation was carried out using G*Power version 3.1.9.6 (Heinrich-Heine-Universität Düsseldorf).^26^

### Statistical Analysis

For baseline characteristics analysis, variables were properly described as means and SDs, medians and IQRs, or counts and frequencies. For normally distributed data, unpaired *t* tests were performed, and for nonnormally distributed data, Mann-Whitney *U* tests were used. χ^2^ or Fisher exact tests were used to analyze categorical variables. All analyses of the RCT were performed on the modified intention-to-treat cohort, defined as all patients who were assigned to treatment and had at least 1 follow-up assessment.

The primary outcome (percentage PANSS score change) was evaluated by a mixed model for repeated measures (MMRM), which is a better approach than both last observation carried forward^27^ and multiple imputation^28^ when dealing with ignorable missing data of clinical trials. The secondary outcome of response to treatment was evaluated using logistic regressions, with dropouts considered as no response or no remission to treatment. Sensitivity analyses were conducted for secondary outcomes. Exploratory outcomes were evaluated analogously to the evaluation of primary outcome. The dose of medications was converted to chlorpromazine equivalents using the R package chlorpromazineR,^29^ with data from the International Consensus Study^30^ as reference.

Statistical significance was defined at a 2-tailed α level of .05. No correction for multiple testing of exploratory outcomes was applied. SPSS Statistics version 26.0 (IBM) and R version 4.2.1 (The R Foundation) were used for statistical analyses. All available postbaseline outcomes measures were included in the analysis.

## Results

### Demographic Characteristics

**Figure 1.**
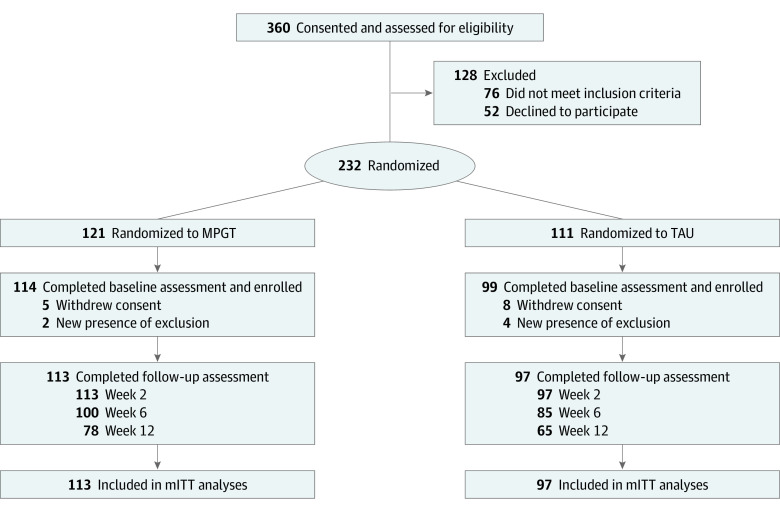
Study Flowchart mITT indicates modified intention to treat; MPGT, multigenetic pharmacogenomics–guided treatment; TAU, treatment as usual.

A total of 360 patients were screened for eligibility, of whom 213 patients entered the trial and 210 were included in the modified intention-to-treat analyses, with 113 randomized to MPGT and 97 to TAU. The mean (SD) age of the 210 included participants was 29.2 (8.8) years, and 63 participants (30.0%) had first-episode schizophrenia. The mean (SD) PANSS score at baseline was 102.1 (14.0). A summary of baseline characteristics is shown in Table 1, and the genotype distribution of participants is shown in eTable 5 in Supplement 2. There were no significant differences in baseline characteristics between treatment groups, except that the MPGT group was more likely to have first-episode schizophrenia and had a shorter duration of illness. A total of 100 patients in the MPGT group (88.5%) and 85 patients in the TAU group (87.6%) completed the 6-week treatment (Figure 1), and there was no difference in rates of completion between groups. A small number of participants received nonpsychiatric concomitant medications during the course of treatment (eTable 6 in Supplement 2).

**Table 1.** Baseline Characteristics of the Modified Intention-to-Treat Population

Characteristic	Patients, No. (%)		
	MPGT (n = 113)	TAU (n = 97)	Total (N = 210)
Age, mean (SD), y	29.9 (8.8)	28.3 (8.8)	29.2 (8.8)
Education, median (IQR), y	9 (9-12)	9 (9-12)	9 (9-12)
Duration of illness, median (IQR), mo	25 (6-72)	36 (13-79)	36 (12-72)
Family history of schizophrenia	7 (6.2)	5 (5.4)	12 (5.8)
Current smoker	34 (30.1)	27 (28.2)	61 (29.0)
First-episode schizophrenia	42 (37.2)	21 (21.6)	63 (30.0)
Baseline PANSS score, mean (SD)	103.5 (14.5)	100.0 (13.2)	102.1 (14.0)
Medication			
Quetiapine	30 (26.5)	19 (19.6)	49 (23.3)
Risperidone	35 (31.0)	44 (45.4)	79 (37.6)
Olanzapine	15 (13.3)	11 (11.3)	26 (12.4)
Aripiprazole	17 (15.0)	8 (8.2)	25 (11.9)
Ziprasidone	1 (0.9)	3 (3.1)	4 (1.9)
Paliperidone	7 (6.2)	7 (7.2)	14 (6.7)
Clozapine	2 (1.8)	2 (2.1)	4 (1.9)
Amisulpride	6 (5.3)	3 (3.1)	9 (4.3)
Metabolic profile			
Triglyceride, median (IQR), mg/dL	103.7 (76.2-162.1)	97.5 (67.3-163.0)	102.8 (75.3-162.1)
Cholesterol, median (IQR), mg/dL	162.3 (129.1-184.3)	145.3 (126.0-172.3)	153.0 (127.6-180.5)
LDL cholesterol, median (IQR), mg/dL	97.4 (75.4-120.6)	92.8 (74.6-121.4)	95.5 (74.6-119.8)
HDL cholesterol, median (IQR), mg/dL	42.5 (34.0-51.4)	39.8 (33.6-47.1)	40.6 (34.0-47.9)
Fasting plasma glucose, median (IQR), mg/dL	93.7 (87.9-102.7)	93.9 (87.2-100.2)	94.8 (88.1-103.2)
Prolactin, median (IQR), ng/mL	29.9 (20.8-37.7)	32.9 (20.2-47.3)	31.5 (20.1-42.8)

Abbreviations: HDL, high-density lipoprotein; LDL, low-density lipoprotein; MPGT, multigenetic pharmacogenomics–guided treatment; PANSS, Positive and Negative Syndrome Scale; TAU, treatment as usual.

SI conversion factor: To convert triglycerides to mmol/L, multiply by 0.0113; cholesterol to mmol/L, multiply by 0.0259; and glucose to mmol/L, multiply by 0.0555.

### Primary Outcome

**Figure 2.**
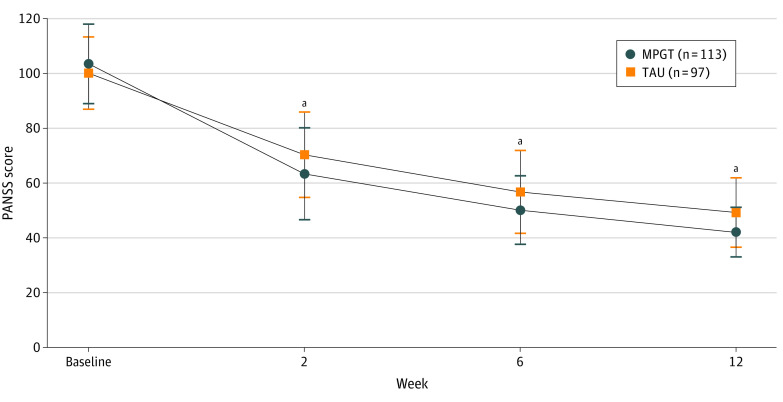
Time Course of Severity Indicated by Positive and Negative Symptom Scale (PANSS) Scores Error bars indicate standard errors. MPGT indicates multigenetic pharmacogenomics–guided treatment; TAU, treatment as usual. ^a^*P* < .01.

**Figure 3.**
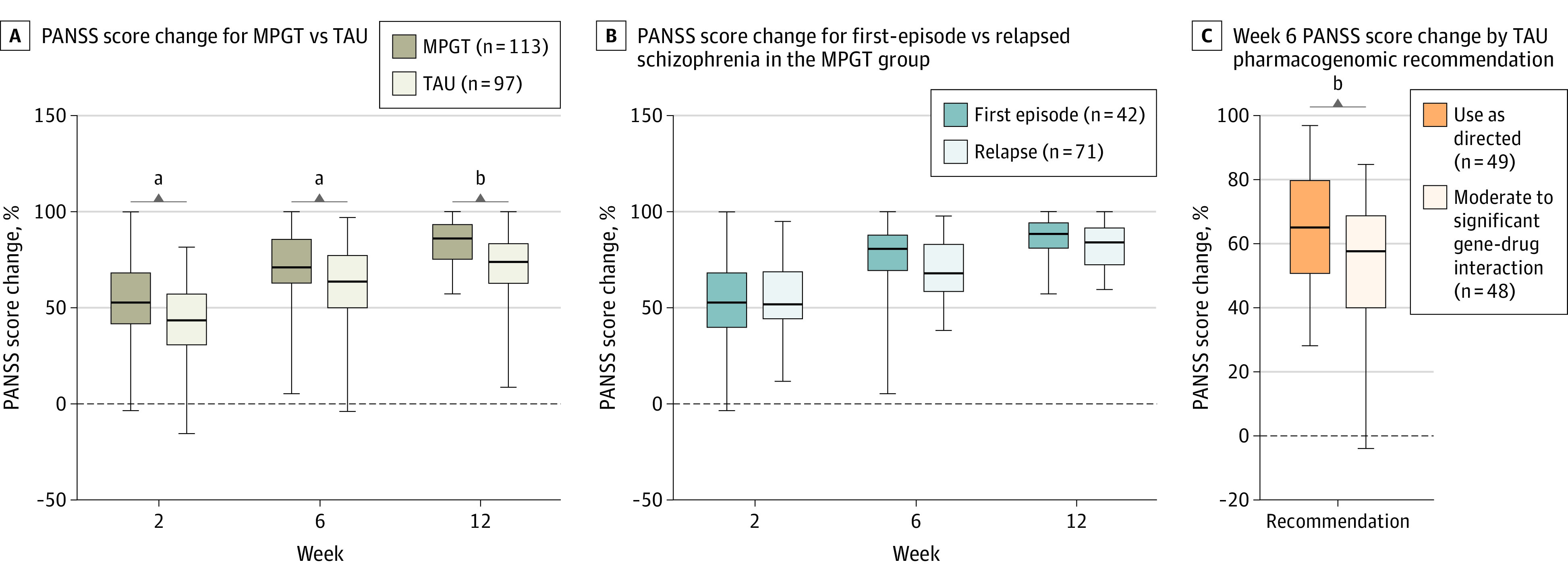
Study Outcome Measures The midline indicates the median; the box, IQRs; and error bars, the range. MPGT indicates multigenetic pharmacogenomics–guided treatment; PANSS, Positive and Negative Syndrome Scale; TAU, treatment as usual. ^a^*P* < .01. ^b^*P* < .001.

The time course of schizophrenia severity represented by PANSS scores and the percentage PANSS score change throughout the whole study are shown in Figure 2 and Figure 3A. The MPGT group showed a significantly higher percentage PANSS score change from baseline to the end of week 6 than the TAU group (74.2% vs 64.9%; least-square mean difference, 9.2 percentage points; 95% CI, 4.4-14.1 percentage points; *P* < .001). Further analysis of differences in percentage PANSS score change from baseline to the end of week 2 (56.9% vs 45.6%; least-square mean difference, 11.3 percentage points; 95% CI, 6.1-16.5 percentage points; *P* < .001) and week 12 (83.5% vs 76.1%; least-square mean difference, 7.4 percentage points; 95% CI, 3.0-11.8 percentage points; *P* = .001) yielded similar results to week 6. Adjustments of MMRM analysis for variables of first-episode schizophrenia and duration of illness, which showed differences between treatment groups at baseline, did not affect the results of the primary outcome (eTable 7 in Supplement 2).

### Secondary Outcomes

The early response rate was not statistically significantly different between treatment groups, as assessed by the odds ratio estimated by the logistic regression model. The response rates at the end of week 6 were 82.3% (93 of 113) for the MPGT group and 64.9% (63 of 97) for the TAU group (adjusted odds ratio, 2.48; 95% CI, 1.28-4.80; *P* = .01). The rates of symptomatic remission at the end of week 12 were 62.8% (71 of 113) for the MPGT group and 45.4% (44 of 97) for the TAU group (adjusted odds ratio, 2.03; 95% CI, 1.11-3.60; *P* = .02) (Table 2). Both sensitivity analyses, one treating dropouts as treatment success and another conducted only in completers, had consistent results (eTable 8 in Supplement 2).

**Table 2.** Secondary Outcomes

Secondary outcome	Patients, No. (%)		Adjusted OR (95% CI)	*P* value
	MPGT (n = 113)	TAU (n = 97)		
Early response^a^	108 (95.6)	87 (90.1)	2.25 (0.71-7.12)	.17
Response^b^	93 (82.3)	63 (64.9)	2.48 (1.28-4.80)	.01
Symptomatic remission^c^	71 (62.8)	44 (45.4)	2.03 (1.11-3.60)	.02

Abbreviations: MPGT, multigenetic pharmacogenomics–guided treatment; OR, odds ratio; TAU, treatment-as-usual.

^a^ Early response was defined as a percentage Positive and Negative Syndrome Scale score change of 20% or more at the end of postrandomized week 2.

^b^ Response was defined as a percentage Positive and Negative Syndrome Scale score change of 50% or more at the end of postrandomized week 6.

^c^ Symptomatic remission was defined as a Positive and Negative Syndrome Scale score of 3 or less on items P1 (delusions), P2 (conceptual disorganization), P3 (hallucinatory behavior), N1 (blunted affect), N4 (passive/apathetic social withdrawal), N6 (lack of spontaneity and flow of conversation), G5 (mannerisms and posturing), and G9 (unusual thought content) at the end of week 12.

### Exploratory Outcomes

The difference in percentage PANSS score change between those with first-episode and relapsed schizophrenia in the MPGT group was not statistically significant, indicating a similar therapeutic effect of MPGT on both first-episode and relapsed schizophrenia (Figure 3B; eTable 9 in Supplement 2). In 97 patients in the TAU group, we found that 49 (50.5%) were assigned medications as use as directed according to the experience of clinical physicians, and 48 (49.5%) were assigned medications with moderate or significant gene-drug interaction. Patients who were assigned medications as use as directed had a significantly greater percentage PANSS score change at the end of week 6 (65.5% vs 55.4%; mean difference, 10.1 percentage points; 95% CI, 2.8-17.4 percentage points; *P* = .008) (Figure 3C). In the MPGT group, all participants used medications categorized as use as directed, since the medications were prescribed based on the pharmacogenomics reports.

We found lower chlorpromazine equivalents were assigned to patients in the MPGT group than the TAU group throughout the study, though not statistically significant at the end of week 6 and week 12. MMRM of metabolic profile analysis showed no significant difference in lipid and glucose metabolism between treatment groups, but plasma prolactin levels were lower in the MPGT group (eTable 10 in Supplement 2). Adjustments of MMRM analysis for the type of medications did not result in changes in results. No severe adverse events were reported during the trial.

## Discussion

To our knowledge, this study is the first RCT to evaluate the therapeutic efficacy of MPGT in Chinese Han patients with schizophrenia. Consistent with our hypotheses, patients with schizophrenia randomized to MPGT achieved greater improvements in symptoms and reported lower plasma prolactin levels.

Several studies have evaluated the effects of single test–guided or limited multigenetic-test–guided pharmacotherapy in schizophrenia.^31,32,33^ These studies mainly focused on cytochrome P450s (CYPs), the major metabolic enzyme system, especially *CYP2D6* and *CYP2C19*. One RCT using routine genetic testing for *CYP2D6* and *CYP2C19* found that CYP-guided therapy had no effects on antipsychotic drug persistence but had a positive effect on measures of hallucination and delusion in the CYP-guided groups.^31^ Another observational study reported improvement in patient outcomes after *CYP2D6* and *CYP2C19* genetic information was provided to patients and physicians.^32^ The third study evaluated the cost-effectiveness of *HLA-DQB1*/*HLA-B* pharmacogenetic-guided treatment in patients with schizophrenia treated with clozapine.^33^ Yet to our knowledge, no studies have evaluated the effects of multigenetic pharmacogenomic tests on treatment outcomes of schizophrenia. However, meta-analyses of RCTs evaluating the effects of multigenetic pharmacogenomic testing–guided antidepressant treatment on major depressive disorder showed that guided treatment was 1.7-fold more likely to attain symptom remission compared with TAU.^34,35^ These studies provided promising positive support for the clinical utility of pharmacogenomics-guided treatment in the field of psychiatry.

In our study, patients treated with MPGT responded better to antipsychotic medications but with relevantly lower doses, which can be seen in the greater reduction of PANSS score and higher response rate. We found that 93 of 113 pharmacogenomics-guided patients (82.3%) reached a 50% reduction of PANSS score at the end of week 6 and 71 of 113 (62.8%) achieved symptomatic remission at the end of week 12. Psychotic symptoms were reported to be significantly associated with patients’ psychosocial functioning, cognitive function, and quality of life,^36,37,38^ and symptomatic remission was related to better overall symptomatic status, better functioning level, better quality of life, and better cognitive performance.^39^ The increase in response rate in our study was significant and clinically meaningful. More interestingly, we found no difference in the MPGT treatment outcome in patients with first-episode and relapsed schizophrenia. Most previous studies on pharmacogenomics have evaluated patients who have not responded to previous treatment, and it remains unclear whether these tests are beneficial among first-episode or drug-naive patients.^40^ Our results may suggest the utility of pharmacogenomic testing in patients with first-episode schizophrenia. However, factors such as cost-effectiveness should be considered in this condition, and as the sample with first-episode schizophrenia was relatively small in our study, the result should be interpreted with caution.

The genetic variants included in the multigenetic pharmacogenomic testing of our study involved drug metabolism, transporters, and receptors. A significant number of the loci detected were also related to CYPs. Most of the psychiatric drugs currently available are metabolized by CYPs, especially CYP1A2, CYP2C19, CYP2D6, and CYP3A4 isoforms.^41^ Genetic variants of CYP enzymes can explain a large proportion of interindividual drug concentration variation, which in turn affects drug therapeutic efficacy and adverse effects. Other genetic variants detected here were related to antipsychotic responses or adverse effects, such as *DRD2* rs1799978^42^ and *EPM2A* rs1415744^43^ for response to olanzapine, and *MC4R* rs489693 for adverse effects to quetiapine and amisulpride.^44^ In our study, we found that about half of patients in the TAU group were using suboptimal medications indicated by the pharmacogenomic testing, indicating that from a pharmacogenomic perspective, it is challenging for physicians to select an optimal medication relying solely on their experience. This might suggest a potential benefit to these patients if pharmacogenomic reports could be provided to physicians before treatment initiation. Apart from potentially reducing prolactin elevation, our results did not find significant benefits of MPGT in reducing adverse effects. This suggests that there may be limitations in the selection of genetic variants related to adverse effects during the design of pharmacogenomic testing. Further research is warranted to address these limitations. Overall, these exploratory results provide more evidence for pharmacogenomic testing in guiding antipsychotic treatments.

### Limitations

There are several limitations of our study. We only included male Chinese Han patients, and the response and remission rate in our study is relatively higher than in previous studies. Caution is needed in generalizing the findings to other populations, especially the treatment-resistant population. Moreover, to choose medications based on the pharmacogenomics report, clinicians were not masked to the study groups, which might increase the clinicians’ attention toward the patient’s medical treatment. Our study was a short-term study, and we did not evaluate the cost-effectiveness of pharmacogenomic testing. Long-term benefits and cost-effectiveness should be investigated in future studies with larger sample sizes and completer assessments.

Evidence regarding the impact of CYP1A2 on the metabolism of antipsychotics is limited and inconsistent. As a result, we only considered its potential influence on clozapine and reduced its weight value. Nevertheless, due to the small number of patients using clozapine in our study, further research is needed to elucidate this matter more comprehensively. Moreover, while we restricted the use of CYP2D6 inhibitors, we did not limit the use of medications that might affect CYP1A2 and CYP3A4 enzymes. Factors such as smoking and coffee consumption, which can affect the activity of the CYP1A2 enzyme, were not accounted for in our study. Additionally, we did not document each nonpsychiatric medication that patients were concurrently taking. These factors must be considered in clinical practice when considering medication selection.

It is important to acknowledge that our pharmacogenomic testing panel was primarily based on East Asian populations and did not encompass all potential loci and variants. For instance, our detection of *CYP2D6* variants covers approximately 86% of all potential variations in East Asian populations, based on published data from PharmGKB,^45,46^ yet it does not include all must-test alleles according to the Association of Molecular Pathology consensus recommendations.^47^ Directly applying our panel in other populations may result in incorrect prediction of CYP2D6 metabolizers, consequently leading to inaccurate reports. This same scenario applies to CYP1A2 and CYP3A4^48^ as well. Therefore, when designing pharmacogenomic testing panels for diverse populations, it is crucial to consider variations in allele frequencies and tailor the design accordingly. Furthermore, we only included a modest number of genes and variants in our testing panel, and there have been ongoing discoveries of new genes and loci associated with the efficacy and adverse effects of antipsychotic medications. These factors should be considered for inclusion in future design of pharmacogenomic testing. In addition, as we did not evaluate the contribution of each gene-drug pair to the treatment outcome, nor did we compare the algorithm in our study to others; it remains unclear whether different algorithms using other gene-drug pairs will work as well.

## Conclusions

In summary, our study showed that pharmacogenomics-guided treatment of antipsychotics significantly improved the clinical outcomes in terms of drug efficacy and adverse effects of prolactin elevation in patients with schizophrenia. Multigenetic pharmacogenomics testing can be an effective tool to guide the treatment of schizophrenia.

## Notes

Supplement 1.
Trial Protocol

Supplement 2. **eMethods.** Multigenetic Pharmacogenomics Testing, Trial Procedure, and Statistical Analysis **eTable 1.** Alleles and Variants Included in the Multigenetic Pharmacogenomics Testing **eTable 2.** Gene-Drug Pairs and Weight Values of Detected Genes and Drugs Used in the Trial **eTable 3.** Example PGx Report **eTable 4.** List of CYP2D6 Inhibitors **eTable 5.** Genotype Distribution of Participants **eTable 6.** Nonpsychiatric Concomitant Medication Use **eTable 7.** Intergroup Comparison of Percentage PANSS Score Change of Follow-Ups **eTable 8.** Sensitivity Analysis of Secondary Outcomes in mITT Cohort Treating Dropouts as Treatment Success and Completers **eTable 9.** Subgroup Analysis of Percentage PANSS Score Change in Patients of MPGT **eTable 10.** Intergroup Comparison of Exploratory Outcome Measurements Across All Follow-Ups
eReferences.

eAppendix.

Supplement 3.
Data Sharing Statement
