# Supplementary material for: Multigenetic Pharmacogenomics–Guided Treatment vs Treatment As Usual Among Hospitalized Men With Schizophrenia: A Randomized Clinical Trial
Source: JAMA Netw Open. 2023 Oct 6;6(10):e2335518. doi: 10.1001/jamanetworkopen.2023.35518 (PMC10559185; doi:10.1001/jamanetworkopen.2023.35518)
Supplement: Supplement 1. — Trial Protocol [file jamanetwopen-e2335518-s001.pdf]

Randomized controlled trials of the therapeutic efficacy and safety of individualized pharmacogenomics-guided treatment in patients with schizophrenia and major depressive disorder

1. Abstract

Schizophrenia and major depressive disorder (MDD) are common mental diseases with a heavy disease burden, and poor compliance of patients is a major problem in the clinical treatment. The objective of this study was to investigate the genetic mechanism of individualized differences in the response to antipsychotic and antidepressant drugs in the Han Chinese population. We plan to enroll 200 patients with schizophrenia and 200 patients with MDD, and randomly assign them to Guided and Unguided treatment groups. Saliva samples will be collected from oral swabs and DNA will be extracted using a commercial pharmacogenomics (PGx) testing kit from Conlight Medical Inc., Shanghai, China. After testing, the PGx reports will be provided to clinicians of the Guided group to guide drug selection, while PGx reports of the Unguided group will be kept until the end of the study. The Unguided group receives treatment-as-usual (TAU). Treatment efficacy and security will be compared between the Guided and Unguided groups, using standardized/quantified clinical records, efficacy information, and genetic information.

2. Background

Individualized medicine takes into consideration of patient's sex, age, weight, physiological and pathological characteristics, genetic factors, and current drug usage, to develop a safe, reasonable, effective, and cost-efficient tailored treatment regimen. A large number of research show that genetic factors are important factors influencing individual differences in drug response, that is, patients' drug response-related gene affect individual therapeutic effects. According to the World Health Organization (WHO), a third of all deaths worldwide are due to inappropriate drug use rather than natural diseases themselves. There are about 50 million inpatients in China every year, of which at least 2.5 million are related to adverse drug reactions, and about 200,000 people die from them, becoming the fourth leading cause of death after cancer and cardiovascular disease. Safe drug use has become a global public health problem, and it is imperative and urgent to carry out individualized drug therapy.

On May 6, 2018, the Mayo Clinic and the National Institutes of Health (NIH) launched the “All of Us Research Program”. European and American countries have developed commercial detection products such as Genesight, Genecepet, and Neuropharmagen for guiding drug therapy. These commercial PGx testing products can increase the response rate of antidepressants and antipsychotics by 2.26 times and the remission rate by 2.5 times; increase compliance by 6.3%, significantly improved the quality of life of patients, reduced the incidence of adverse drug reactions, and reduced the cost of treatment of $3,764 per person per year. Some of these testing have been included in the European and American national health insurance directory.

Research on genomics, personalized medicine, and common psychiatric diseases has made rapid progress recently. At present, international neuroscience research is developing rapidly. Global consortium for genomic research on mental disorders, including the PGC, BROAD, and other research consortiums or institutions, has shown a partial monopoly, which is difficult for domestic academic institutions and other international institutions to compete with. The large global cohort of schizophrenia genomics and pharmacogenomics studies has identified a large number of genetic variants associated with individual differences in disease pathogenesis, drug efficacy, and adverse reactions. With the development of pharmacogenomics in clinical research, the first gene testing platform has been approved in the United States to detect CYP2D6 and CYP2C19, which makes up for the shortage of individualized drug administration only based on blood drug

concentration in the past. Therefore, clinicians can optimize the drug administration plan according to the individual genetic characteristics of patients, and truly achieve personalized medication, to bring efficient, safe, and cost-efficient treatment for patients with mental illness. In addition, functional studies on schizophrenia susceptibility genes are in progress. The regulation mechanism of the signaling pathway of these genes remains to be explored. Elucidating the biological functions of these genetic variants will help to find biomarkers for the prevention, diagnosis, drug therapy, and prognosis of mental diseases and the target of drug therapy.

According to the literature, about 66.5% of patients with schizophrenia did not achieve clinical response after taking antipsychotics (PANSS reduction rate <50%); About 66.9% of the patients did not achieve clinical remission. Antipsychotics have a 30-50% discontinuation rate due to poor efficacy. Meta-analyses of clinical studies over the past 30 years have shown that the clinical cure rate of antidepressants is 30-40%. Studies have shown that genetic factors account for approximately 42-50% of individual differences in response and adverse reactions to antipsychotics and antidepressants, providing a basis for individualized medication for psychiatric disorders.

There are a variety of antipsychotics and antidepressants. Proper selection of drugs, dose, treatment course, and rational combination of these drugs can achieve the best efficacy and the least adverse reactions. However, the scientific research on how to guide the proper use of these drugs is far behind the actual clinical needs. Therefore, in a sense, how to choose the appropriate drug treatment regimen for patients is a clinical skill. In recent years, many domestic companies have launched gene testing projects, starting from the gene level to design individualized drug therapy, and finally, realize individualized drug use from "treating the disease" to "treating the person". The research on the susceptibility genes of schizophrenia and MDD in the Chinese population still needs to be improved. Finding the susceptibility genes of schizophrenia and MDD in the Chinese population and verifying their correlation with susceptibility genes will help guide the development of targeted drugs and provide an important basis for the individualized treatment of schizophrenia and MDD.

3. Objective and relevance

The purpose of this study is to explore the mechanisms of the inheritance of individual differences in response to antipsychotics and antidepressants. We plan to enroll 200 cases of schizophrenia and 200 cases of MDD in China and use the PGx testing from Conlight Medical that was approved by State Food and Drug Administration (SFDA). Participants will be randomly assigned to accept PGx-guided treatment or TAU.

Using standardized/quantified clinical cases, efficacy information, and genetic studies, the relationship between the efficacy of antipsychotics and the genes/loci associated with response or adverse reactions in Han Chinese was further examined.

4. Research procedure

4.1 Specific Objectives

Based on the pharmacogenomics studies of schizophrenia and MDD in the Chinese Han population and internationally, a randomized controlled trial is designed to explore the efficacy and safety of individualized PGx-guided treatment of schizophrenia and MDD, compared with current conventional treatment. To further identify the genes/loci associated with the efficacy and adverse reactions of antipsychotics and antidepressants in Han Chinese and their potential functions, and provide clues for the individualized treatment of schizophrenia and MDD.

4.2 A randomized controlled trial of the therapeutic efficacy and safety of individualized pharmacogenomics-guided treatment in patients with

schizophrenia

4.2.1 Study design

A 12-week, two-center, patient- and rater-blinded, parallel, randomized, controlled trial evaluating the therapeutic effects of individualized PGx-guided treatment in patients with SCH, with a comparison to a TAU group. The total number of participants will be 200 (100 per group). Participants will be assessed at baseline, the end of week 2, 6, and 12.

4.2.2 Participant recruitment

(1) Sample size: 200

(2) Inclusion criteria: Inpatient, Han nationality, gender unlimited, 18-60 years old; can provide written informed consent signed by patient or guardian; diagnosed with schizophrenia by 2 attending psychiatrists according to the *Diagnostic and Statistical Manual of Mental Disorders, Fourth Edition* (*DSM-IV*) criteria for schizophrenia, have a total score ≥60 of the Positive and Negative Syndrome Scale (PANSS) at baseline. The patient should have a fixed residence, be able to complete a 12-week follow-up of antipsychotic therapy, and agree to cooperate with the investigator to complete the follow-up treatment on time.

(3) Exclusion criteria: Suffering from severe physical diseases (such as metabolic diseases, kidney diseases, liver diseases, thyroid diseases, etc.) or organic brain diseases, which can refer to having a deviation more than twice from the normal value of laboratory tests, or refer to the research doctor's opinion; a history of loss of consciousness for more than 5 minutes; a history of alcohol or substance abuse/dependence within the past 1 year; electroconvulsive therapy in the past six months; regular use of addictive drugs within one month; presence of other mental disorders (personality disorders and mental retardation) that significantly affect the patient's current mental state; women who is pregnant or breastfeeding, or who plans to become pregnant; other situation that researchers consider incompliant with the research.

(4) Withdrawal and termination criteria: Presence of any of the above exclusion criteria, any severe adverse events, or upon patient request.

4.2.3 Randomization and masking

Patients enrolled will be randomly assigned to the individualized PGx-guided treatment group or the TAU group in a 1:1 ratio using a pre-planned randomization list containing 200 random numbers. According to the order in which they were enrolled, each patient will be assigned a random number, with an odd number standing for the guided group, and an even number for TAU. The table of random numbers is generated by authorized personnel from the Evidence-Based Medicine Centre of Peking University Sixth Hospital, using the *Formula* function of Excel, =INT(200*RAND()+1). Raters are blinded to the study arm. Patients are blinded to the study arm and their PGx report until the end of the trial. Clinicians who take care of patients in the guided group have an access to the PGx report to guide drug selection.

4.2.4 Interventions

Participants first complete all baseline assessments and laboratory tests. Within the first week, all patients receive a low dose of risperidone ≤2 mg/d (an equivalent dose conversion of other antipsychotics), and benzodiazepines are allowed to use as treatments for agitation or insomnia if required. At the end of the first week, when PGx reports are available, patients in the guided group receive drug therapy according to the reports, and patients in the TAU group receive drug therapy according to the clinicians' experience. Participants complete 2-, 6- and 12-weeks follow-up, which include comprehensive physical examination, laboratory tests (blood routine test, blood biochemistry test), auxiliary tests (electrocardiogram, electroencephalogram), clinical scale evaluation (PANSS, CGI), etc.

4.2.5 Measurement

The primary efficacy endpoint of this trial was the percentage PANSS change from the baseline to week 6. Percentage PANSS change is calculated as:

$$\triangle P = (PANSS_0 - PANSS_6) \times 100\% / (PANSS_0 - 30)$$

$PANSS_0$ indicates the total PANSS score at baseline, and $PANSS_6$ indicates the total PANSS score at the end of week 6.

The secondary endpoint was response or remission rates at each time point. Early response was defined as a percentage PANSS change of ≥ 20% at week 2, response was defined as a percentage PANSS change of ≥ 50%, and symptomatic remission was defined as a PANSS score of ≤ 3 on items P1, P2, P3, N1, N4, N6, G5, and G9 at week 12.

Use the clinical global impression (CGI) Scale as supplemental evaluation of clinical efficacy.

4.3 A randomized controlled trial of the therapeutic efficacy and safety of individualized pharmacogenomics-guided treatment in patients with MDD

4.3.1 Study design

An 8-week, two-center, patient- and rater-blinded, parallel, randomized, controlled trial evaluating the therapeutic effects of individualized PGx-guided treatment in patients with MDD, with a comparison to a TAU group. The total number of participants will be 200 (100 per group). Participants will be assessed at baseline, the end of week 4 and 8.

4.3.2 Participant recruitment

(1) Sample size: 200

(2) Inclusion criteria: Han nationality, gender unlimited, 18-55 years old; can provide written informed consent signed by patient or guardian; diagnosed with MDD by 2 attending psychiatrists according to the *Diagnostic and Statistical Manual of Mental Disorders, Fourth Edition* (*DSM-IV*) criteria for MDD, have a total score ≥ 18 of the 17-item Hamilton Depression Scale (HAMD-17) at baseline; require oral medication treatment. The patient should have a fixed residence, be able to complete an 8-week follow-up of antidepressants therapy, and agree to cooperate with the investigator to complete the follow-up treatment on time.

(3) Exclusion criteria: Diagnosed with other mental disorders according to DSM-IV criteria, were pregnant or lactating, regularly used addictive drugs for nearly one month, had significant risk for suicide and severe or unstable physical diseases, had experienced more than 5 minutes of loss of consciousness or had received electroconvulsive therapy in the past six months.

(4) Withdrawal and termination criteria: Presence of any of the above exclusion criteria, any severe adverse events, or upon patient request.

4.3.3 Randomization and masking

Patients enrolled will be randomly assigned to the individualized PGx-guided treatment group or the TAU group in a 1:1 ratio using a pre-planned randomization list containing 200 random numbers. According to the order in which they were enrolled, each patient will be assigned a random number, with an odd number standing for the guided group, and an even number for TAU. The table of random numbers is generated by authorized personnel from the Evidence-Based Medicine Centre of Peking University Sixth Hospital, using the *Formula* function of Excel, =INT(200*RAND()+1). Raters are blinded to the study arm. Patients are blinded to the study arm and their PGx report until the end of the trial. Clinicians who take care of patients in the guided group have an access to the PGx report to guide drug selection.

4.3.4 Interventions

Participants first complete all baseline assessments and laboratory tests. Within the first week, all patients receive a low dose of escitalopram (≤ 10 mg/d) or an equivalent dose conversion of other antidepressants, and benzodiazepines are allowed to use as treatments for agitation or insomnia if required. At the end of the first week, when PGx reports are available, patients in the guided group receive drug therapy according to the reports, and patients in the TAU group receive drug therapy according to the clinicians’ experience. Participants complete 4- and 8-weeks follow-up, which include

comprehensive physical examination, laboratory tests (blood routine test, blood biochemistry test), auxiliary tests (electrocardiogram, electroencephalogram), clinical scale evaluation (HAMD-17, HAMA), etc.

4.3.5 Measurement

The primary efficacy endpoint of this trial was the percentage HAMD change from the baseline to week 8. Percentage HAMD change is calculated as:

$$\text{HAMD percentage change} = 100\% * \frac{\text{Baseline Score} - \text{Follow up Score}}{\text{Baseline Score}}$$

The secondary endpoint was response or remission rates at each time point, with response defined as a ≥ 50% reduction in HAMD-17 scores and remission defined as HAMD-17 scores ≤ 7.

5. Genetic testing

Using matrix-assisted laser desorption-ionization time of flight mass spectrometry (MALDI-TOF MS) gene analysis system. PGx testing will be carried out by Conlight Medical.

Antipsychotic related genes are as follows: *ABCB1*, *ANKK1*, *COMT*, *CYP1A2*, *CYP2D6*, *CYP3A4*, *DRD2*, *EPM2A*, *GNB3*, *HTR1A*, *HTR2A*, *HTR2C*, *MC4R*, *RGS4*, *SH2B1*, *SLC6A4*, etc

Antidepressant related genes are as follows: CYP2C19, CYP2D6, CYP1A2, CYP2B6, ABCB1, SLC6A4, HTR1A, HTR2A, FKBP5, etc.

6. Statistical analysis

The main outcome analyses will be conducted according to the “intent-to-treat” principle. The primary endpoint will be evaluated by a mixed model for repeated measures (MMRM), with the assumption that data are missing at random. The latter assumption will be tested. MMRM will be analyzed with treatment group, study center, time point of assessment as a categorical variable, baseline PANSS/HAMD-17 sum score, and time × treatment group interaction included in the model as fixed effects, while patient-specific effects enter the model as a random effect. Other potentials will also be included in the model according to the data distribution. An unstructured covariance matrix for the residuals will be used. The analyses will be supplemented by cross-sectional comparisons. Effect sizes will be calculated with Cohen’s *d.* Statistical significance was defined at an alpha level of 0.05 by two-tailed.

7. Data Confidentiality

All the work described in this protocol will be carried out in China, and all specimens collected for this study will be destroyed after analysis. The study data will be stored at Peking University Sixth Hospital. The study does not involve the export of human genetic resources/information.

All the samples and data collected in this project will be used for long-term research in our laboratory, except for the oral pharyngeal subsamples for one-time detection. In accordance with the legal requirements, we strictly keep the confidentiality of participants’ research records, unless at the request of law. The participant's name, address, telephone, or any information that can directly identify their identity will not be leaked to the team. Cooperation units that participate in this study have no access to the biological samples and genetic information of the original data. When information and data from this study are presented at scientific meetings or in scientific journals, each participant will be identified by a unique number, and their identity will not be disclosed. Encoding information will be kept properly.
