# Supplementary material for: Multigenetic Pharmacogenomics–Guided Treatment vs Treatment As Usual Among Hospitalized Men With Schizophrenia: A Randomized Clinical Trial
Source: JAMA Netw Open. 2023 Oct 6;6(10):e2335518. doi: 10.1001/jamanetworkopen.2023.35518 (PMC10559185; doi:10.1001/jamanetworkopen.2023.35518)
Supplement: Supplement 2. — eMethods. Multigenetic Pharmacogenomics Testing, Trial Procedure, and Statistical Analysis eTable 1. Alleles and Variants Included in the Multigenetic Pharmacogenomics Testing eTable 2. Gene-Drug Pairs and Weight Values of Detected Genes and Drugs Used in the Trial eTable 3. Example PGx Report eTable 4. List of CYP2D6 Inhibitors eTable 5. Genotype Distribution of Participants eTable 6. Nonpsychiatric Concomitant Medication Use eTable 7. Intergroup Comparison of Percentage PANSS Score Change of Follow-Ups eTable 8. Sensitivity Analysis of Secondary Outcomes in mITT Cohort Treating Dropouts as Treatment Success and Completers eTable 9. Subgroup Analysis of Percentage PANSS Score Change in Patients of MPGT eTable 10. Intergroup Comparison of Exploratory Outcome Measurements Across All Follow-Ups eReferences. eAppendix. [file jamanetwopen-e2335518-s002.pdf]

Supplemental Online Content

Kang Z, Qin Y, Sun Y, et al. Multigenetic pharmacogenomics–guided treatment vs treatment as usual among hospitalized men with schizophrenia: a randomized clinical trial. *JAMA Netw Open*. 2023;6(10):e2335518. doi:10.1001/jamanetworkopen.2023.35518

eMethods. Multigenetic Pharmacogenomics Testing, Trial Procedure, and Statistical Analysis
eTable 1. Alleles and Variants Included in the Multigenetic Pharmacogenomics Testing
eTable 2. Gene-Drug Pairs and Weight Values of Detected Genes and Drugs Used in the Trial
eTable 3. Example PGx Report
eTable 4. List of CYP2D6 Inhibitors
eTable 5. Genotype Distribution of Participants
eTable 6. Nonpsychiatric Concomitant Medication Use
eTable 7. Intergroup Comparison of Percentage PANSS Score Change of Follow-Ups
eTable 8. Sensitivity Analysis of Secondary Outcomes in mITT Cohort Treating Dropouts as Treatment Success and Completers
eTable 9. Subgroup Analysis of Percentage PANSS Score Change in Patients of MPGT
eTable 10. Intergroup Comparison of Exploratory Outcome Measurements Across All Follow-Ups
eReferences.
eAppendix.

This supplementary material has been provided by the authors to give readers additional information about their work.

© 2023 Kang Z et al. *JAMA Network Open*.

eMethods. Multigenetic Pharmacogenomics Testing, Trial Procedure, and Statistical Analysis

Multigenetic pharmacogenomics testing

In this study, we detected single nucleotide polymorphism (SNP) loci of 26 alleles or variants across 11 genes that are reported to be associated with antipsychotic medication metabolism, efficacy, or side effects (eTable 1). The selection and interpretation of these detected genes and variants were based on publicly available data, including published literature, pharmGKB[1], the Clinical Pharmacogenetics Implementation Consortium (CPIC) database (https://cpicpgx.org/), U.S. Food and Drug Administration (FDA) (https://www.fda.gov/), and European Medicines Agency (EMA) (https://www.ema.europa.eu/). For the sake of testing expense, convenience, and the complexity of interpretation, we chose to concentrate on genes associated with treatment efficacy or adverse reactions for multiple medications, omitting those related to only one or two medications. Subsequently, we screened the variants in our previously collected cohort to identify potential loci relevant to antipsychotic treatment outcomes in the Han Chinese population. These selected loci were then incorporated into our PGx testing panel, and their weight values concerning treatment efficacy were assigned according to their effect sizes observed in our cohort. A total of 8 commonly used atypical antipsychotics, namely amisulpride, aripiprazole, clozapine, olanzapine, paliperidone, quetiapine, risperidone, and ziprasidone were included in our study.
Genomic DNA was isolated from buccal samples, and genotyping examinations were conducted by Conlight Medical Inc. (Shanghai, China), using the MassArray (MALDI-TOF MS) genotyping method. Subsequently, we employed an algorithm, based on licensed technologies disclosed in issued patents (CHN patent no. CN109777870B and CHN patent no. CN115295116A, pss-system.cponline.cnipa.gov.cn, eAppendix), to evaluate the combined effects of the tested variants and generate reports with medication selection recommendations. In this algorithm, each drug is matched with genes or SNPs associated with its metabolism, efficacy, or side effects. Different weight values were assigned to variations of each gene or SNP matched (eTable 2). The weight values for variations related to therapeutic efficacy is based on the effect sizes observed in our screening cohort, while all variations that contribute to an increased occurrence of adverse reactions are assigned a value of 1. In the assignment of weight values to variations of metabolism-related genes, we followed the Dutch Pharmacogenetics Working Group (DPWG) guideline[2,3]. According to the guidelines, genotype variations with significant evidence of influencing drug metabolism are assigned a weight of 2, while variations with limited evidence receive a weight of 1 or 0.5. The sum of the variation intensities was calculated using the formula $V_{drug} = weight_{SNP1} + weight_{SNP2} + ... + weight_{SNPn}$. Different PGx recommendation levels were determined based on the value of V_{drug}. If $V_{drug} < 1$, the drug is categorized as “Use as Directed”, indicating a minimal or no gene-drug interaction, allowing physicians to use the drug directly in appropriate circumstances. If $1 \leq V_{drug} < 2$, the drug is categorized as “Moderate Gene-drug Interaction” and should be used after evaluation. If $V_{drug} \geq 2$, the drug is categorized as “Significant Gene-drug Interaction” and should be used under blood concentration monitoring or consider alternations (eTable 3 for an example PGx report).

Trial procedure

At baseline, the *Chinese version of Structured Clinical Interview for the DSM-IV-TR Axis I Disorders*[4] (SCID) was used to determine the diagnoses of patients. Patients who met the inclusion criteria were randomized into two groups (MPGT and TAU). Then general information and clinical characteristics were collected, and baseline clinical laboratory tests of metabolic profile were assessed. Clinical symptoms were assessed using the Positive and Negative Syndrome Scale (PANSS)[5]. After that, all patients had the multigenetic PGx testing and received a low-dose treatment (≤ 2 mg/d) of risperidone or an equivalent dose conversion of other antipsychotic medication for one week.
At the end of the first week (when the PGx report was available), patients in the MPGT group received antipsychotic medications recommended by the report of PGx testing. Clinicians chose medications placed in the category of “Use as Directed”, or if no drugs were in this category, drugs placed in the category of “Moderate Gene-drug Interaction”. Patients in the TAU group received antipsychotic medications according to the experience of clinical physicians. Benzodiazepines were allowed to use as treatments for agitation or insomnia if

© 2023 Kang Z et al. *JAMA Network Open*.

required. Other psychotropic drugs, such as antidepressants and mood stabilizers, were not allowed. The use of non-psychiatric medications was not restricted, except for those that have significant interactions with antipsychotics (e.g., an inhibitor of the CYP2D6 enzyme). To be more specific, we provided a list of CYP2D6 inhibitors (eTable 4) to the physicians, and any medications not on the list were required to be checked against drug package inserts to confirm whether they are CYP2D6 inhibitors. Antipsychotics were not allowed to be switched in the MPGT group. However, for ethical reasons, clinicians could switch antipsychotics in the TAU group when participants showed insufficient response or intolerance to antipsychotics that have been used.

At the end of week 2, 6, and 12 after the treatment assignment, follow-up assessments of PANSS and clinical laboratory tests were conducted to evaluate the treatment outcome. The dose of medications was also recorded. Follow-up assessments were conducted by raters who were blinded to the treatment assignment. Non-psychiatric concomitant medication use was also recorded (eTable 6).

Statistical analysis

The primary outcome was evaluated by a mixed model for repeated measures (MMRM). We included treatment group, study center, time point of assessment, baseline PANSS sum score, and time × treatment group interaction in the model as fixed effects, while patient-specific effects enter the model as a random effect. An unstructured covariance matrix for the residuals was used. The analyses were supplemented by cross-sectional comparisons. Least squares estimate for the two treatment groups as well as for differences between the two treatment groups at each time point are reported with 95% confidence intervals and P values.

The secondary outcome of response to treatment was evaluated using logistic regressions, with dropouts as no response/remission to treatment. We included treatment group, study center, and first-episode schizophrenia in the logistic regression model as factors, and baseline PANSS sum score as well as the duration of illness as a covariate. Treatment effects are reported as adjusted odds ratios with 95% confidence intervals and P values. We conducted two sensitivity analyses to the secondary outcome, with one treating dropouts as treatment success, and another excluding dropout.

The exploratory outcomes of dose of medications and metabolic profile was analyzed similar to the primary outcome by MMRM, which included treatment group, study center, time point of assessment, baseline PANSS sum score, and time × treatment group interaction in the model as fixed effects, while patient-specific effects enter the model as a random effect. For the comparison of the differences in treatment efficacy among different recommendation levels in the TAU group, unpaired *t* test of LOCF populations was used.

© 2023 Kang Z et al. *JAMA Network Open*.

eTable 1. Alleles and Variants Included in the Multigenetic Pharmacogenomics Testing

**Gene (number of variants)**	**Alleles/Variants evaluated**
CYP1A2 (4)	*1, *1C, *1F, *6
CYP2D6 (9)	*1, *10, *2, *2A, *4, *5, *14A, *14B, *41, CNV
CYP3A4 (2)	*1, *20
DRD2 (3)	rs1799978, rs1799732, rs1079597
EPM2A (1)	rs1415744
HTR1A (1)	rs10042486
HTR2A (1)	rs7997012
HTR2C (2)	rs1414334, rs6318
MC4R (1)	rs489693
RGS4 (1)	rs951439
SH2B1 (1)	rs3888190

Abbreviations: CNV, copy number variants; *CYP1A2,* Cytochrome P450 Family 1 Subfamily A Member 2; *CYP2D6,* Cytochrome P450 Family 2 Subfamily D Member 6; *CYP3A4,* Cytochrome P450 Family 3 Subfamily A Member 4; *DRD2,* Dopamine Receptor D2; *EPM2A,* EPM2A Glucan Phosphatase, Laforin; *HTR1A*, 5-Hydroxytryptamine Receptor 1A; *HTR2A,* 5-Hydroxytryptamine Receptor 2A; *HTR2C*, 5-Hydroxytryptamine Receptor 2C; *MC4R*, Melanocortin 4 Receptor; *RGS4,* Regulator of G Protein Signaling 4; *SH2B1*, SH2B Adaptor Protein 1.

© 2023 Kang Z et al. *JAMA Network Open*.

eTable 2. Gene-Drug Pairs and Weight Values of Detected Genes and Drugs Used in the Trial

Drug	Metabolism/PK	Efficacy			Side effects		
**Amisulpride**	/	*DRD2* rs1079597[6]	*HTR1A* rs10042486[7]		*SH2B1* rs3888190[8]	*MC4R* rs489693[9,10]	
		CT (0.5)	CC (0.5)		CC (0)	AA (1)	
		CC (0)	CT (0.5)		AC (1)	AC (0)	
		TT (0)	TT (0)		AA (1)	CC (0)	
**Aripiprazole**	*CYP2D6* metabolizer[2,3]	*DRD2* rs1799732[11-13]			*SH2B1* rs3888190[8]	*MC4R* rs489693[9]	
	PM (2)	del/del (1)			CC (0)	AA (1)	
	UM (0.5)	G/del (1)			AC (1)	AC (0)	
	IM (0.5)	GG (0)			GG (1)	CC (0)	
	NM (0)	/			/	/	
**Clozapine**	*CYP1A2* metabolizer[14,15]	*DRD2* rs1799732[11,16]			*SH2B1* rs3888190[8]	*MC4R* rs489693[9,10]	*HTR2C* rs1414334[17-21]
	PM (1)	del/del (1)			CC (0)	AA (1)	C (1)
	UM (1)	G/del (1)			AC (1)	AC (0)	G (0)
	IM (0.5)	GG (0)			AA (1)	CC (0)	/
	NM (0)	/			/	/	/
**Olanzapine**	/	*DRD2* rs1799978[22]	*EPM2A* rs1415744[23]	*RGS4* rs951439[24]	*HTR2A* rs7997012[25]	*MC4R* rs489693[10]	
		CC (0.5)	CC (0.5)	TT (0.5)	AA (1)	AA (1)	
		CT (0.5)	CT (0.5)	CT (0)	GG (0)	AC (0)	
		TT (0)	TT (0)	CC (0)	AG (0)	CC (0)	
**Quetiapine**	*CYP3A4* metabolizer[2,3]	*HTR1A* rs10042486[7]	*RGS4* rs951439[24]	*EPM2A* rs1415744[23]	*MC4R* rs489693[9]		
	PM (2)	CT (0.5)	CC (0.5)	TT (0.5)	AA (1)		
	UM (0.5)	CC (0.5)	CT (0)	CT (0)	AC (0)		
	IM (0.5)	TT (0)	TT (0)	TT (0)	CC (0)		
	NM (0)	/	/	/	/		
**Paliperidone**	/	/			*MC4R* rs489693[10]	*SH2B1* rs3888190[26]	
					AA (1)	CC (1)	
					AC (0)	AC (0)	
					CC (0)	AA (0)	
**Risperidone**	*CYP2D6* metabolizer[2,3]	*HTR1A* rs10042486[7]			*SH2B1* rs3888190[8]	*MC4R* rs489693[9,10,27]	
	PM (2)	CT (1)			CC (0)	AA (1)	
	UM (2)	CC (1)			AC (1)	AC (0)	
	IM (0.5)	TT (0)			AA (1)	CC (0)	
	NM (0)	/			/	/	
**Ziprasidone**	/	*RGS4* rs951439[24]			*MC4R* rs489693[9]		
		CC (0.5)			AA (1)		
		CT (0)			AC (0)		
		TT (0)			CC (0)		

Note: Numbers in parentheses represent the weight values.
Abbreviations: PK, pharmacokinetics; *CYP2D6,* Cytochrome P450 Family 2 Subfamily D Member 6; *CYP3A4,* Cytochrome P450 Family 3 Subfamily A Member 4; *DRD2,* Dopamine Receptor D2; *EPM2A,* EPM2A Glucan Phosphatase, Laforin; *HTR1A*, 5-Hydroxytryptamine Receptor 1A; *HTR2A,* 5-Hydroxytryptamine Receptor 2A; *HTR2C*, 5-Hydroxytryptamine Receptor 2C; *MC4R*, Melanocortin 4 Receptor; *RGS4,* Regulator of G Protein Signaling 4; *SH2B1*, SH2B Adaptor Protein 1; IM, intermediate metabolizer; NM, normal metabolizer; PM, poor metabolizer; UM, ultrarapid metabolizer.

eTable 3. Example PGx Report

Use as Directed	Moderate Gene-drug Interaction	Significant Gene-drug Interaction
Amisulpride[5, 7]<br>Risperidone[1, 5, 7]<br>Ziprasidone[5, 7]<br>Paliperidone[7]	Olanzapine[5, 8]<br>Aripiprazole[1, 5, 8]	Clozapine[2, 5, 8]<br>Quetiapine[4, 6, 8]

Note:
1. (Normal metabolizer) Drug metabolism is normal and blood drug concentration within the treatment window.
2. (Ultrarapid metabolizer) Drug metabolism is accelerated and blood concentration may be lower than effective treatment concentration, leading to poor treatment effect.
3. (Intermediate metabolizer) Drug metabolism is slowed down, and blood concentration may be higher than safe therapeutic concentration (or lack of clear data to support).
4. (Poor metabolizer) Drug metabolism is significantly slowed, and blood concentrations may be higher than safe therapeutic concentrations, leading to an increased risk of adverse effects.
5. Predictive drug response is relatively good.
6. Predictive drug response is relatively poor.
7. Lower risk of adverse effects.
8. Higher risk of adverse effects.

© 2023 Kang Z et al. *JAMA Network Open*.

eTable 4. List of CYP2D6 Inhibitors

CYP2D6 inhibitors
Buspirone, amiodarone, warfarin, chlorpheniramine, cimetidine, diphenhydramine, hydroxyzine, propafenone, quinidine, ritonavir

© 2023 Kang Z et al. *JAMA Network Open*.

eTable 5. Genotype Distribution of Participants

Gene (SNP)	Genotype / Metabolizer	Patients, No. (%)		
		MPGT (n = 113)	TAU (n = 97)	Total (n = 210)
CYP1A2	UM	64 (56.6)	60 (61.9)	124 (59.0)
	NM	48 (42.5)	37 (38.1)	85 (40.5)
	IM	1 (0.9)	0 (0)	1 (0.5)
CYP2D6	UM	3 (2.7)	0 (0)	3 (1.4)
	NM	60 (53.1)	47 (48.5)	107 (51.0)
	IM	49 (43.4)	48 (49.5)	97 (46.2)
	PM	1 (0.9)	2 (2.1)	3 (1.4)
CYP3A4	EM	113 (100)	97 (100)	210 (100)
DRD2 (rs1799978)	TT	68 (60.2)	56 (57.7)	124 (59.0)
	CT	38 (33.6)	37 (38.1)	75 (35.7)
	CC	7 (6.2)	4 (4.1)	11 (5.2)
DRD2 (rs1799732)	GG	98 (86.7)	83 (85.6)	181 (86.2)
	G/DEL	13 (11.5)	12 (12.4)	25 (11.9)
	DEL/DEL	2 (1.8)	2 (2.1)	4 (1.9)
DRD2 (rs1079597)	CC	48 (42.5)	41 (42.3)	89 (42.4)
	CT	43 (38.1)	39 (40.2)	82 (39.0)
	TT	22 (19.5)	17 (17.5)	39 (18.6)
EPM2A (rs1415744)	CC	70 (61.9)	60 (61.9)	130 (61.9)
	CT	35 (31.0)	29 (29.9)	64 (30.5)
	TT	8 (7.1)	8 (8.2)	16 (7.6)
HTR1A (rs10042486)	TT	83 (73.5)	57 (58.8)	140 (66.7)
	CT	28 (24.8)	37 (38.1)	65 (31.0)
	CC	2 (1.8)	3 (3.1)	5 (2.4)
HTR2A (rs7997012)	GG	55 (48.7)	48 (49.5)	103 (49.0)
	AG	55 (48.7)	40 (41.2)	95 (45.2)
	AA	3 (2.7)	9 (9.3)	12 (5.7)
HTR2C (rs1414334)	GG	113 (100)	95 (97.9)	208 (99.0)
	CC	0 (0)	2 (2.1)	2 (1.0)
HTR2C (rs6318)	GG	113 (100)	95 (97.9)	208 (99.0)
	CC	0 (0)	2 (2.1)	2 (1.0)
MC4R (rs489693)	CC	88 (77.9)	61 (62.9)	149 (71.0)
	AC	25 (22.1)	28 (28.9)	53 (25.2)
	AA	0 (0)	8 (8.2)	8 (3.8)
RGS4 (rs951439)	CC	29 (25.7)	25 (25.8)	54 (25.7)
	CT	67 (59.3)	52 (53.6)	119 (56.7)
	TT	17 (15.0)	20 (20.6)	37 (17.6)
SH2B1 (rs3888190)	CC	93 (82.3)	65 (67.0)	158 (75.2)
	AC	18 (15.9)	30 (30.9)	48 (22.9)
	AA	2 (1.8)	2 (2.1)	4 (1.9)

Abbreviations: *CYP2D6,* Cytochrome P450 Family 2 Subfamily D Member 6; *CYP3A4,* Cytochrome P450 Family 3 Subfamily A Member 4; *DRD2,* Dopamine Receptor D2; *EPM2A,* EPM2A Glucan Phosphatase, Laforin; *HTR1A*, 5-Hydroxytryptamine Receptor 1A; *HTR2A,* 5-Hydroxytryptamine Receptor 2A; *HTR2C*, 5-Hydroxytryptamine Receptor 2C; *MC4R*, Melanocortin 4 Receptor; *RGS4,* Regulator of G Protein Signaling 4; *SH2B1*, SH2B Adaptor Protein 1; IM, intermediate metabolizer; NM, normal metabolizer; PM, poor metabolizer; UM, ultrarapid metabolizer.

© 2023 Kang Z et al. *JAMA Network Open*.

eTable 6. Nonpsychiatric Concomitant Medication Use

Time points	Patients, No./total No. (%)		
	**MPGT**	**TAU**	**Total**
Baseline	1/113 (0.9)	2/97 (1.0)	3/210 (1.4)
Week 2	3/113 (2.7)	5/97 (5.2)	8/210 (3.8)
Week 6	3/100 (3.0)	5/85 (5.9)	8/185 (4.3)
Week 12	3/78 (3.8)	1/65 (1.5)	4/143 (2.8)

Abbreviations: MPGT, multigenetic pharmacogenomics-guided treatment; TAU, treatment as usual.

© 2023 Kang Z et al. *JAMA Network Open*.

eTable 7. Intergroup Comparison of Percentage PANSS Score Change of Follow-Ups

Time points	Mean (95% CI)		Mean difference (95% CI)	*P* value
	MPGT (n = 113)	TAU (n = 97)		
MMRM[a]				
Week 2	56.9 (52.7 to 61.1)	45.6 (41.0 to 50.2)	11.3 (6.1 to 16.5)	<.001
Week 6	74.2 (70.2 to 78.1)	64.9 (60.2 to 69.3)	9.2 (4.4 to 14.1)	<.001
Week 12	83.5 (80.0 to 87.0)	76.1 (72.2 to 80.0)	7.4 (3.0 to 11.8)	.001
MMRM adjusted for additional variables[b]				
Week 2	57.2 (52.8 to 61.5)	46.2 (41.2 to 51.1)	11.0 (5.7 to 16.3)	<.001
Week 6	74.3 (70.3 to 78.4)	65.6 (61.0 to 70.3)	8.7 (3.7 to 13.7)	<.001
Week 12	83.7 (80.0 to 87.4)	76.6 (72.3 to 80.9)	7.1 (2.6 to 11.6)	.002

Abbreviations: MPGT, multigenetic pharmacogenomics-guided treatment; TAU, treatment as usual; PANSS, Positive and Negative Syndrome Scale; MMRM, mixed model for repeated measures.

[a] MMRM included treatment group, study center, time point of assessment, baseline PANSS sum score, and time × treatment group interaction in the model as fixed effects, while patient-specific effects enter the model as a random effect.

[b] MMRM further adjusted for first-episode schizophrenia and duration of illness.

© 2023 Kang Z et al. *JAMA Network Open*.

eTable 8. Sensitivity Analysis of Secondary Outcomes in mITT Cohort Treating Dropouts as Treatment Success and Completers

Secondary outcomes	Patients, No. (%)		Adjusted odds radio for MPGT vs TAU (95% CI)	P value
	MPGT (n = 113)	TAU (n = 97)		
mITT cohort treating dropouts as treatment success				
Early response[a]	108 (95.6)	87 (90.1)	2.25 (0.71 to 7.12)	.17
Response[b]	106 (93.8)	75 (77.3)	4.11 (1.60 to 10.54)	.003
Symptomatic Remission[c]	105 (92.9)	76 (78.4)	4.55 (1.71 to 12.09)	.002
Completers[d]				
Early response[a]	77 (98.7)	55 (84.6)	45.09 (2.53 to 802.32)	.01
Response[b]	76 (97.4)	46 (70.8)	18.71 (3.69 to 94.82)	< .001
Symptomatic Remission[c]	71 (91.0)	44 (67.7)	6.54 (2.23 to 19.20)	.001

Abbreviations: MPGT, multigenetic pharmacogenomics-guided treatment; TAU, treatment as usual.

[a] Early response was defined as a percentage PANSS change of ≥ 20% at the end of post-randomized week 2.

[b] Response was defined as a percentage PANSS change of ≥ 50% at the end of post-randomized week 6.

[c] Symptomatic remission was defined as a PANSS score of ≤ 3 on items P1, P2, P3, N1, N4, N6, G5, and G9 at the end of week 12.

[d] MPGT, n = 78; TAU, n = 65.

© 2023 Kang Z et al. *JAMA Network Open*.

eTable 9. Subgroup Analysis of Percentage PANSS Score Change in Patients of MPGT

Time points	Mean (95% CI)		Mean difference (95% CI)	*P* value
	First-episode (n = 42)	Relapsed (n = 71)		
Week 2	57.2 (49.8 to 64.6)	56.5 (50.8 to 62.3)	0.65 (-7.25 to 8.56)	.87
Week 6	77.5 (71.4 to 83.5)	72.2 (67.4 to 76.9)	5.30 (-1.09 to 11.69)	.10
Week 12	85.7 (80.6 to 90.7)	81.8 (77.8 to 85.8)	3.83 (-1.45 to 9.11)	.15

Abbreviations: MPGT, multigenetic pharmacogenomics-guided treatment; PANSS, Positive and Negative Syndrome Scale.

© 2023 Kang Z et al. *JAMA Network Open*.

eTable 10. Intergroup Comparison of Exploratory Outcome Measurements Across All Follow-Ups

Outcome measurements	Mean (95% CI)		Mean difference (95% CI)	*P* value
	MPGT (n = 113)	TAU (n = 97)		
Chlorpromazine equivalent, mg/d				
Week 2	435 (410 to 460)	485 (458 to 544)	-50 (-87 to -13)	.008
Week 6	463 (430 to 496)	509 (474 to 544)	-46 (-94 to 2)	.06
Week 12	407 (374 to 441)	456 (419 to 493)	-49 (-99 to 1)	.05
Triglyceride, mg/dL				
Week 2	169.2 (145.3 to 193.1)	170.1 (143.5 to 195.8)	-0.9 (-30.1 to 28.3)	.96
Week 6	178.0 (149.7 to 206.4)	171.0 (139.9 to 202.0)	-7.09 (-29.2 to 43.4)	.71
Week 12	186.9 (152.3 to 222.3)	195.8 (156.8 to 235.6)	-8.8 (-54.0 to 36.3)	.69
Cholesterol, mg/dL				
Week 2	170.9 (162.4 to 179.8)	167.0 (157.3 to 176.3)	4.2 (-6.57 to 14.6)	.45
Week 6	175.1 (165.8 to 184.4)	170.1 (160.0 to 180.2)	4.6 (-6.5 to 16.2)	.41
Week 12	178.2 (167.8 to 188.7)	167.8 (156.2 to 179.4)	10.5 (-2.71 to 23.5)	.11
LDL cholesterol, mg/dL				
Week 2	109.8 (103.6 to 115.6)	103.6 (97.0 to 110.6)	5.8 (-1.55 to 14.6)	.11
Week 6	110.9 (104.4 to 117.9)	106.3 (99.0 to 113.6)	4.6 (-3.48 to 12.7)	.27
Week 12	117.5 (109.8 to 124.9)	112.1 (103.6 to 120.2)	5.5 (-3.87 to 14.6)	.25
HDL cholesterol, mg/dL				
Week 2	45.6 (40.1 to 51.0)	49.1 (43.0 to 54.9)	-3.4 (-10.2 to 3.4)	.32
Week 6	46.4 (40.4 to 52.2)	47.2 (40.8 to 53.7)	-0.9 (-8.5 to 6.5)	.80
Week 12	41.7 (37.6 to 46.0)	46.4 (41.8 to 51.0)	-4.7 (-10.0 to 0.6)	.08
FPG, mg/dL				
Week 2	90.8 (87.4 to 94.0)	91.2 (87.5 to 94.9)	-0.5 (-4.7 to 3.6)	.82
Week 6	95.3 (91.9 to 98.5)	98.2 (94.6 to 101.8)	-2.9 (-7.0 to 1.3)	.16
Week 12	98.4 (93.3 to 103.4)	104.5 (98.9 to 110.3)	-6.1 (-12.6 to 0.4)	.06
Prolactin, ng/mL				
Week 2	35.5 (28.4 to 42.7)	52.6 (45.0 to 60.2)	-17.0 (-25.8 to -8.2)	<.001
Week 6	28.8 (21.9 to 35.7)	45.6 (38.1 to 53.2)	-16.8 (-25.8 to -8.2)	<.001
Week 12	29.4 (21.7 to 37.0)	40.4 (31.9 to 48.9)	-11.0 (-20.8 to -1.3)	.03

Abbreviations: MPGT, multigenetic pharmacogenomics-guided treatment; TAU, treatment as usual; PANSS, Positive and Negative Syndrome Scale; LDL, low-density lipoprotein; HDL, high-density lipoprotein; FPG, fasting plasma glucose.
Note: All results are based on MMRM estimates.

© 2023 Kang Z et al. *JAMA Network Open*.

eReferences.

1. Whirl-Carrillo M, Huddart R, Gong L, et al. An Evidence-Based Framework for Evaluating Pharmacogenomics Knowledge for Personalized Medicine. *Clin Pharmacol Ther*. Sep 2021;110(3):563-572. doi:10.1002/cpt.2350
2. Beunk L, Nijenhuis M, Soree B, et al. Dutch Pharmacogenetics Working Group (DPWG) guideline for the gene-drug interaction between CYP2D6, CYP3A4 and CYP1A2 and antipsychotics. *Eur J Hum Genet*. Mar 31 2023;(1476-5438 (Electronic))doi:10.1038/s41431-023-01347-3
3. Swen JJ, Nijenhuis M, de Boer A, et al. Pharmacogenetics: from bench to byte--an update of guidelines. *Clin Pharmacol Ther*. May 2011;89(5):662-73. doi:10.1038/clpt.2011.34
4. Glasofer DR, Brown AJ, Riegel M. Structured Clinical Interview for DSM-IV (SCID). *Encyclopedia of Feeding and Eating Disorders*. 2015:1-4:chap Chapter 80-1.
5. Kay SR, Fiszbein A, Opler LA. The positive and negative syndrome scale (PANSS) for schizophrenia. *Schizophr Bull*. 1987;13(2):261-76. doi:10.1093/schbul/13.2.261
6. Kang SG, Na KS, Lee HJ, Chee IS, Lee K, Lee J. DRD2 genotypic and haplotype variation is associated with improvements in negative symptoms after 6 weeks' amisulpride treatment. *J Clin Psychopharmacol*. Apr 2015;35(2):158-62. doi:10.1097/JCP.0000000000000294
7. Crisafulli C, Chiesa A, Han C, et al. Case-control association study for 10 genes in patients with schizophrenia: influence of 5HTR1A variation rs10042486 on schizophrenia and response to antipsychotics. *Eur Arch Psychiatry Clin Neurosci*. Apr 2012;262(3):199-205. doi:10.1007/s00406-011-0278-3
8. Delacretaz A, Zdralovic A, Vandenberghe F, et al. Association of variants in SH2B1 and RABEP1 with worsening of low-density lipoprotein and glucose parameters in patients treated with psychotropic drugs. *Gene*. Sep 10 2017;628(1879-0038 (Electronic)):8-15. doi:10.1016/j.gene.2017.07.005
9. Malhotra AK, Correll CU, Chowdhury NI, et al. Association between common variants near the melanocortin 4 receptor gene and severe antipsychotic drug-induced weight gain. *Arch Gen Psychiatry*. Sep 2012;69(9):904-12. doi:10.1001/archgenpsychiatry.2012.191
10. Czerwensky F, Leucht S, Steimer W. MC4R rs489693: a clinical risk factor for second generation antipsychotic-related weight gain? *Int J Neuropsychopharmacol*. Oct 2013;16(9):2103-9. doi:10.1017/S1461145713000849
11. Zhang JP, Lencz T, Malhotra AK. D2 receptor genetic variation and clinical response to antipsychotic drug treatment: a meta-analysis. *Am J Psychiatry*. Jul 2010;167(7):763-72. doi:10.1176/appi.ajp.2009.09040598
12. Shen YC, Chen SF, Chen CH, et al. Effects of DRD2/ANKK1 gene variations and clinical factors on aripiprazole efficacy in schizophrenic patients. *J Psychiatr Res*. Mar 2009;43(6):600-6. doi:10.1016/j.jpsychires.2008.09.005
13. Eum S, Schneiderhan ME, Brown JT, Lee AM, Bishop JR. Pharmacogenetic evaluation to assess breakthrough psychosis with aripiprazole long-acting injection: a case report. *BMC Psychiatry*. Jul 3 2017;17(1):238. doi:10.1186/s12888-017-1396-x
14. Lesche D, Mostafa S, Everall I, Pantelis C, Bousman CA. Impact of CYP1A2, CYP2C19, and CYP2D6 genotype- and phenoconversion-predicted enzyme activity on clozapine

© 2023 Kang Z et al. *JAMA Network Open*.

exposure and symptom severity. *Pharmacogenomics J*. Apr 2020;20(2):192-201. doi:10.1038/s41397-019-0108-y
15. Doude van Troostwijk LJ, Koopmans RP, Vermeulen HD, Guchelaar HJ. CYP1A2 activity is an important determinant of clozapine dosage in schizophrenic patients. *Eur J Pharm Sci*. Dec 2003;20(4-5):451-7. doi:10.1016/j.ejps.2003.09.010
16. Hwang R, Shinkai T, De Luca V, et al. Association study of 12 polymorphisms spanning the dopamine D(2) receptor gene and clozapine treatment response in two treatment refractory/intolerant populations. *Psychopharmacology (Berl)*. Aug 2005;181(1):179-87. doi:10.1007/s00213-005-2223-5
17. Mulder H, Franke B, van der-Beek van der AA, et al. The association between HTR2C gene polymorphisms and the metabolic syndrome in patients with schizophrenia. *J Clin Psychopharmacol*. Aug 2007;27(4):338-43. doi:10.1097/JCP.0b013e3180a76dc0
18. Risselada AJ, Vehof J, Bruggeman R, et al. Association between HTR2C gene polymorphisms and the metabolic syndrome in patients using antipsychotics: a replication study. *Pharmacogenomics J*. Feb 2012;12(1):62-7. doi:10.1038/tpj.2010.66
19. Mulder H, Cohen D, Scheffer H, et al. HTR2C gene polymorphisms and the metabolic syndrome in patients with schizophrenia: a replication study. *J Clin Psychopharmacol*. Feb 2009;29(1):16-20. doi:10.1097/JCP.0b013e3181934462
20. Ma X, Maimaitirexiati T, Zhang R, et al. HTR2C polymorphisms, olanzapine-induced weight gain and antipsychotic-induced metabolic syndrome in schizophrenia patients: a meta-analysis. *Int J Psychiatry Clin Pract*. Oct 2014;18(4):229-42. doi:10.3109/13651501.2014.957705
21. Rico-Gomis JM, Palazon-Bru A, Triano-Garcia I, et al. Association between the HTR2C rs1414334 C/G gene polymorphism and the development of the metabolic syndrome in patients treated with atypical antipsychotics. *PeerJ*. 2016;4:e2163. doi:10.7717/peerj.2163
22. Lencz T, Robinson DG, Xu K, et al. DRD2 promoter region variation as a predictor of sustained response to antipsychotic medication in first-episode schizophrenia patients. *Am J Psychiatry*. Mar 2006;163(3):529-31. doi:10.1176/appi.ajp.163.3.529
23. Porcelli S, Balzarro B, Lee SJ, et al. PDE7B, NMBR and EPM2A Variants and Schizophrenia: A Case-Control and Pharmacogenetics Study. *Neuropsychobiology*. 2016;73(3):160-8. doi:10.1159/000445295
24. Campbell DB, Ebert PJ, Skelly T, et al. Ethnic stratification of the association of RGS4 variants with antipsychotic treatment response in schizophrenia. *Biol Psychiatry*. Jan 1 2008;63(1):32-41. doi:10.1016/j.biopsych.2007.04.018
25. Laika B, Leucht S, Heres S, Schneider H, Steimer W. Pharmacogenetics and olanzapine treatment: CYP1A2*1F and serotonergic polymorphisms influence therapeutic outcome. *Pharmacogenomics J*. Feb 2010;10(1):20-9. doi:10.1038/tpj.2009.32
26. Delacretaz A, Zdralovic A, Vandenberghe F, et al. Association of variants in SH2B1 and RABEP1 with worsening of low-density lipoprotein and glucose parameters in patients treated with psychotropic drugs. *Gene*. Sep 10 2017;628:8-15. doi:10.1016/j.gene.2017.07.005
27. Nurmi EL, Spilman SL, Whelan F, et al. Moderation of antipsychotic-induced weight gain by energy balance gene variants in the RUPP autism network risperidone studies. *Transl Psychiatry*. Jun 25 2013;3(6):e274. doi:10.1038/tp.2013.26

© 2023 Kang Z et al. *JAMA Network Open*.

eAppendix.

Note: This supplementary material provides the English translated version of the patent referenced in the manuscript (CHN patent no. CN115295116A, available at pss-system.cponline.cnipa.gov.cn).

Title of Invention
A method, system, and electronic device for medication evaluation

Abstract
The present application provides a method, system, and electronic device for medication evaluation related to the field of bioinformatics technology. The method includes obtaining a patient's sample information, which includes disease information and genetic testing results; preprocessing the genetic testing results, which include gene loci and genotypes; identifying multiple first gene loci and their corresponding first genotypes, and obtaining auxiliary interpretation genes and type interpretation results based on the disease information; matching treatment drugs based on the disease information, identifying first auxiliary interpretation gene and/or the second gene locus, and the first type interpretation results corresponding to the first auxiliary interpretation genes and/or the second genotypes corresponding to the second gene loci are interpreted to obtain medication interpretation results. A personalized medication evaluation plan is generated by combining all medication interpretation results to provide reference and assistance for decision-making.

Claims
1. A medication evaluation method, comprising: obtaining a patient's sample information, which includes disease information and genetic testing results, preprocessing the genetic testing results, which include gene loci and genotypes correspond to the gene loci; based on the disease information, identifying and interpreting multiple first gene loci and their corresponding first genotypes to obtain auxiliary interpretation genes and type interpretation results, where the type interpretation results correspond one-to-one with the auxiliary interpretation genes; based on the disease information, matching treatment drugs, identifying the first auxiliary interpretation gene and/or the second gene locus associated with the treatment drug, searching for the first type interpretation result corresponding to the first auxiliary interpretation gene, identifying the second genotypes corresponding to the second gene loci, and interpreting the first type interpretation results and/or the second genotypes to obtain medication interpretation results of the treatment drug; generating a personalized medication evaluation plan by combining all medication interpretation results to provide reference and assistance for decision-making.

© 2023 Kang Z et al. *JAMA Network Open*.

2. The method according to claim 1, characterized in that the preprocessing of the obtained gene detection results includes: repeating the formatting of the gene detection results to generate multiple formatted entries; determining whether the formatted entries are compliant; if the formatted entries are compliant, outputting the formatted entries as genetic detection result entries and storing them, wherein the gene detection result entry includes the patient's sample number, the gene locus, and the corresponding genotype of the gene locus.

3. The method according to claim 1, characterized in that the identification and interpretation of multiple first gene loci and corresponding first genotypes based on disease information to obtain auxiliary interpretation genes and type interpretation results includes: determining the auxiliary interpretation gene based on the disease information; identifying multiple first gene loci and corresponding first genotypes based on the auxiliary interpretation genes; interpreting all the first gene loci and corresponding first genotypes of the auxiliary interpretation genes to obtain the corresponding type interpretation results.

4. The method according to claim 1, characterized in that the treatment drug interpretation result is obtained by interpreting the first type interpretation results and/or the second genotypes, comprising: preprocessing the gene detection results, including: repeating the format processing of the genetic detection results and generating multiple processed entries; determining whether the processed entries are compliant; if the processed entries are compliant, outputting the processed entries as genetic detection result entries and storing them, including the patient's sample number, the gene locus, and the genotype corresponding to the gene locus; obtaining the basic feature value and weight value of the first auxiliary interpretation gene based on the first auxiliary interpretation gene and the first type interpretation result; generating the comprehensive feature value of the treatment drug based on the basic feature values and weight values of each first auxiliary interpretation gene of the treatment drug through a comprehensive feature value model; and the drug interpretation result includes the comprehensive feature value, comprehensive medication advice, and recommendation level.

5. The method according to claim 1, characterized in that the treatment drug interpretation result is obtained by interpreting the first type interpretation result and/or the second genotype, comprising: obtaining the basic feature value and weight value of the second gene locus based on the second gene locus and the second genotype; generating the comprehensive feature value of the treatment drug based on the basic feature values and weight values of each second gene locus of the treatment drug through a comprehensive feature value model; obtaining comprehensive medication advice and recommendation level based on the comprehensive feature value; and the drug interpretation result includes the comprehensive feature value, comprehensive medication advice, and recommendation level.

© 2023 Kang Z et al. *JAMA Network Open*.

6. The method according to claim 1, characterized in that the drug interpretation result of the treatment drug is obtained by interpreting the first type interpretation result and/or the second genotype, comprising: obtaining the basic feature value and weight value of the first auxiliary interpretation gene based on the first auxiliary interpretation gene and the first type interpretation result; obtaining the basic feature value and weight value of the second gene locus based on the second gene locus and the second genotype; generating the comprehensive feature value of the treatment drug by using the basic feature value and weight value of each first auxiliary interpretation gene and each second gene locus of the treatment drug through a comprehensive feature value model; obtaining the comprehensive medication suggestion and recommendation level based on the comprehensive feature value; and the drug interpretation result comprises the comprehensive feature value, the comprehensive medication suggestion, and the recommendation level.
7. The method according to any one of claims 4 to 6, characterized in that the recommendation level of the treatment drug is determined as prioritized recommended, generally recommended, or cautiously recommended according to the interval in which the comprehensive feature value of the treatment drug falls; different intervals correspond to different recommendation levels of different treatment drugs, or the same intervals correspond to different recommendation levels of different treatment drugs.
8. A medication evaluation system, comprising: an acquisition module for obtaining patient sample information, including disease information and genetic testing results, and preprocessing the genetic testing results, which include gene loci and genotypes that correspond to each other one by one; an auxiliary interpretation module for identifying and reading multiple first gene loci and their corresponding first genotypes based on the disease information, obtaining auxiliary interpretation genes and type interpretation results, which correspond one-to-one with the type interpretation results; an recognition module for matching treatment drugs based on the disease information, identifying first auxiliary interpretation genes and/or second gene loci associated with the treatment drugs, finding first type interpretation results corresponding to the first auxiliary interpretation genes, and identifying second genotypes corresponding to the second gene loci; a medication interpretation module for reading the first type interpretation results and/or the second genotypes to obtain medication interpretation results for the treatment drugs; and a plan generation module for generating personalized medication evaluation plans by combining medication interpretation results for multiple treatment drugs, providing references and assisting decisions.
9. An electronic device, comprising: a processor; and a memory for storing computer-executable instructions, which, when executed, cause the processor to perform any one of the methods according to claims 1-7.

© 2023 Kang Z et al. *JAMA Network Open*.

10. A computer-readable storage medium storing one or more programs that, when executed by a processor, implement any one of the methods according to claims 1-7.

Instruction Manual

A method, system, and electronic device for medication evaluation

Technical Field

[0001] The present invention relates to the field of bioinformatics technology, particularly to a medication evaluation method, system, and electronic device.

Background Art

[0002] Currently, the methods used for genotype data interpretation usually correspond genotype data to medication recommendations based on predetermined interpretation logic and detection data from predetermined loci, through manual interpretation. However, this approach relies on manual operations, which results in low output capacity and efficiency, weak confidentiality, and insufficient accuracy and repeatability due to manual factors.

[0003] Therefore, a medication evaluation method, system, and electronic device are proposed.

Summary of the Invention

[0004] The present manual provides a medication evaluation method, system, and electronic device. By obtaining patient sample information and generating a personalized clinical prescription medication evaluation plan based on the results of two readings of medication interpretations combined with treatment medication interpretations, the present invention provides a reference and assists doctors in making prescription decisions.

[0005] The medication evaluation method provided in this application adopts the following technical solution, including:

[0006] Obtaining patient sample information, including disease information and genetic testing results, preprocessing the genetic testing results, including gene loci and genotypes, and corresponding each gene locus to its genotype;

[0007] Based on the disease information, identifying and interpreting multiple first gene loci and their corresponding first genotypes, and obtaining auxiliary interpretation gene and type interpretation results corresponding to each other.

[0008] Based on the disease information, matching the treatment drugs and identifying the first auxiliary interpretation gene and/or the second gene locus related to the treatment drugs, searching for the first type interpretation result corresponding to the first auxiliary interpretation gene, and identifying the second genotype corresponding to the second gene locus.

[0009] Reading the first type interpretation result and/or the second genotype to obtain the drug interpretation result of the treatment drugs;

[0010] Combining all the drug interpretation results of the treatment drugs to generate a personalized medication evaluation plan for reference and decision-making assistance.
[0011] Optionally, preprocessing the gene detection results, including:
[0012] Repeating the formatting of the gene detection results to generate multiple formatted entries;
[0013] Determining whether the formatted entries are compliant:
[0014] If the formatted entries are compliant, output the formatted entries as genetic detection result entries and store them, which include the patient's sample number, the gene locus, and the genotype corresponding to the gene locus.
[0015] Optionally, identifying and reading multiple first gene loci and their corresponding first genotypes based on the disease information, including:
[0016] Determining the auxiliary interpretation genes based on the disease information:
[0017] Identifying multiple first gene loci and their corresponding first genotypes based on the auxiliary interpretation gene;
[0018] Determine the entire first gene locus and first genotype of the auxiliary interpretation gene to obtain the type interpretation result corresponding to the auxiliary interpretation gene.
[0019] Optionally, obtain the drug interpretation result for the treatment drug based on the first type interpretation result and/or the second genotype, including:
[0020] Obtain the basic feature value and weight value of the first auxiliary interpretation gene based on the first auxiliary interpretation gene and the first type interpretation result.
[0021] Generate the comprehensive feature value of the treatment drug by synthesizing the basic feature value and weight value of each first auxiliary interpretation gene of the treatment drug through a comprehensive feature value model.
[0022] Obtain the comprehensive medication recommendation and recommendation level based on the comprehensive feature value.
[0023] The drug interpretation result includes the comprehensive feature value, comprehensive medication recommendation, and recommendation level.
[0024] Optionally, obtain the drug interpretation result of the treatment drug based on the first type interpretation result and/or the second genotype, including:
[0025] Obtain the basic feature value and weight value of the second gene locus based on the second gene locus and second genotype.
[0026] Generate the comprehensive feature value of the treatment drug by synthesizing the basic feature value and weight value of each second gene locus of the treatment drug through a comprehensive feature value model.

© 2023 Kang Z et al. *JAMA Network Open*.

[0027] Obtain the comprehensive medication recommendation and recommendation level based on the comprehensive feature value.
[0028] The drug interpretation result includes the comprehensive feature value, comprehensive medication recommendation, and recommendation level.
[0029] Optionally, obtain the drug interpretation result of the treatment drug based on the first type interpretation result and/or the second genotype, including:
[0030] Obtain the basic feature value and weight value of the first auxiliary interpretation gene based on the first auxiliary interpretation gene and the first type interpretation result.
[0031] Obtain the basic feature value and weight value of the second gene locus based on the second gene locus and second genotype.
[0032] Generate the comprehensive feature value of the treatment drug by synthesizing the basic feature value and weight value of each first auxiliary interpretation gene and each second gene locus of the treatment drug through a comprehensive feature value model.
[0033] Based on the comprehensive features, comprehensive medication recommendations and recommendation levels are obtained.
[0034] The medication interpretation results include the comprehensive features, comprehensive medication recommendations, and recommendation levels.
[0035] Optionally, based on the interval of the comprehensive features, the recommended level of the treatment drug is determined as one of prioritized recommended, generally recommended, and cautiously recommended. Different intervals of comprehensive features correspond to different recommended levels of different treatment drugs.
[0036] The medication evaluation system provided in this application adopts the following technical scheme, including:
[0037] Acquisition module, used to obtain patient's sample information, including disease information and genetic testing results. The genetic testing results are preprocessed, and the genetic loci correspond one-to-one with the genotypes.
[0038] Auxiliary interpretation module, used to identify and interpret multiple first genetic loci and their corresponding first genotypes based on disease information, and obtain auxiliary interpretation genes and type interpretation results. The type interpretation results correspond one-to-one with the auxiliary interpretation genes.
[0039] Recognition module, used to match treatment drugs based on the disease information, identify the first auxiliary interpretation genes and/or second genetic loci related to the treatment drugs, and search for the corresponding first type interpretation results based on the first auxiliary interpretation genes. The second genotype corresponding to the second genetic loci is identified.

[0040] Medication interpretation module, used to interpret the first type interpretation results and/or the second genotype, and obtain medication interpretation results for the treatment drugs.
[0041] Plan generation module, used to generate personalized medication evaluation plans based on multiple drug interpretation results for reference and decision-making assistance.
[0042] Optionally, the acquisition module includes:
[0043] Formatting sub-module, used to format the genetic test results repeatedly and generate multiple formatting entries;
[0044] Judgment sub-module, used to judge whether the formatting entries are compliant;
[0045] Output sub-module, used to output the formatting entries as genetic test result entries and store them if the formatting entries are compliant, and the genetic test result entries include the patient's sample number, gene locus, and the corresponding genotype.
[0046] Optionally, the auxiliary interpretation module includes:
[0047] Auxiliary gene determination sub-module, used to determine the auxiliary interpretation gene based on the disease information;
[0048] Recognition sub-module, used to recognize multiple first gene loci and their corresponding first genotypes based on the auxiliary interpretation gene;
[0049] Auxiliary interpretation sub-module, used to read all first gene loci and first genotypes corresponding to the auxiliary interpretation gene, and obtain the corresponding type interpretation results.
[00501] Optional, the drug interpretation module includes:
[0051] First acquisition sub-module, used to obtain the basic feature value and weight value of the first auxiliary interpretation gene based on the first auxiliary interpretation gene and the first type interpretation result;
[00521] First feature value generation sub-module, used to generate the comprehensive feature value of the treatment drug by synthesizing the basic feature values and weight values of each first auxiliary interpretation gene of the treatment drug through a comprehensive feature value model;
[0053] First recommendation sub-module, used to obtain comprehensive medication suggestions and recommendation levels based on the comprehensive feature value;
[0054] The drug interpretation result includes the comprehensive feature value, comprehensive medication suggestions, and recommendation level of the treatment drug.
[0055] Optional, the drug interpretation module includes:
[0056] Second acquisition sub-module, used to obtain the basic feature value and weight value of the second gene locus based on the second gene locus and the second genotype;
[0057] Second feature value generation sub-module, used to generate the comprehensive feature value of the treatment drug by synthesizing the basic

feature values and weight values of each second gene locus of the treatment drug through a comprehensive feature value model;
[0058] Second recommendation sub-module, used to obtain comprehensive medication suggestions and recommendation levels based on the comprehensive feature value;
[0059] The drug interpretation result includes the comprehensive feature value, comprehensive medication suggestions, and recommendation level of the treatment drug.
[0060] Optionally, the drug interpretation module includes:
[0061] The third acquisition sub-module, which is used to obtain the basic feature values and weight values of the third auxiliary interpretation gene based on the first auxiliary interpretation gene and the first type interpretation result;
[0062] The fourth acquisition submodule, which is used to obtain the basic feature values and weight values of the second gene locus based on the second gene locus and the second genotype;
[0063] The third feature value generation sub-module, which is used to generate the comprehensive feature value of the treatment drug based on the basic feature values and weight values of each of the first auxiliary interpretation gene and each of the second gene loci of the treatment drug through a comprehensive feature value model;
[0064] The third recommendation sub-module, which is used to obtain the comprehensive medication advice and recommendation level based on the comprehensive feature value;
[0065] The drug interpretation result includes the comprehensive feature value, the comprehensive medication advice, and the recommendation level.
[0066] Optionally, based on the interval in which the comprehensive feature value is located, the recommendation level of the treatment drug is determined to be either prioritized, generally recommended, or cautiously recommended. The interval in which the comprehensive feature value corresponding to the recommendation level of different treatment drugs is located is different.
[0067] This manual also provides an electronic device, which includes:
[0068] A processor; and,
[0069] A memory that stores computer-executable instructions, wherein the processor executes any of the above methods when the executable instructions are executed.
[0070] This manual also provides a computer-readable storage medium, wherein the computer-readable storage medium stores one or more programs, and when the one or more programs are executed by a processor, the processor implements any of the methods described above.
[0071] In this application, by obtaining patient sample information including disease information and genetic test results, wherein the genetic test results include gene loci and genotypes, preprocessing of the genetic test results is

© 2023 Kang Z et al. *JAMA Network Open*.

performed. Based on the disease information, multiple first gene loci and their corresponding first genotypes are identified and read, and auxiliary interpretation genes and type reading results are obtained to improve the reading efficiency of genes or gene loci related to the treatment drugs. Matching the treatment drugs based on the disease information, the first auxiliary interpretation gene and/or the second gene locus related to the treatment drugs are identified, and the first type interpretation results corresponding to the first auxiliary interpretation gene are searched. The second genotype corresponding to the second gene locus is identified. By reading the first type interpretation results and/or the second genotype, the drug interpretation results for the treatment drugs are obtained. Combining all of the drug interpretation results for the treatment drugs, a personalized medication evaluation plan is generated to provide reference and assistance for decision-making.

Description of Figures

[0072] Figure 1 is a schematic diagram illustrating the principle of a medication evaluation method provided in an embodiment of the present manual;

[0073] Figure 2 is a flowchart illustrating the preprocessing of genetic testing results in a medication evaluation method provided in an embodiment of the present manual;

[0074] Figure 3 is a schematic diagram illustrating the structure of a medication evaluation system provided in an embodiment of the present manual;

[0075] Figure 4 is a schematic diagram illustrating the structure of an electronic device provided in an embodiment of the present manual;

[0076] Figure 5 is a schematic diagram illustrating the principle of a computer-readable medium provided in an embodiment of the present manual.

Detail Description

[0077] The following description is provided to disclose the invention so that those skilled in the art can implement the invention. The embodiments described below are for illustration only, and those skilled in the art may conceive of other obvious modifications. The basic principles of the invention defined in the following description can be applied to other embodiments, variations, improvements, equivalent embodiments, and other technical solutions that do not depart from the spirit and scope of the invention.

[0078] Now, exemplary embodiments of the invention will be described in more detail with reference to the accompanying figures. However, the exemplary embodiments can be implemented in various forms, and should not be construed as limiting the invention to the embodiments described herein. Instead, providing these exemplary embodiments can make the invention more comprehensive and complete, and more easily convey the

© 2023 Kang Z et al. *JAMA Network Open*.

inventive concept to those skilled in the art. The same reference numerals in the figures indicate the same or similar elements, components, or portions, and thus, redundant descriptions thereof will be omitted.
[0079] Features, structures, characteristics, or other details described in a particular embodiment may be combined in a suitable manner with one or more other embodiments, while conforming to the technical concept of the invention.
[0080] In describing specific embodiments, features, structures, characteristics, or other details of the invention disclosed herein are included to enable those skilled in the art to fully understand the embodiments. However, it should be understood that those skilled in the art can practice the technical solutions of the present invention without specific features, structures, characteristics, or other details described in a particular embodiment or more embodiments.
[0081] The flowchart shown in the figures is exemplary and not intended to include all content and operations/steps, nor is it necessary to perform the operations/steps in the order described. For example, some operations/steps may be decomposed, and some operations/steps may be combined or partially combined, so the actual execution order may change depending on the actual situation.
[0082] The block diagram shown in the figures is merely a functional entity and does not necessarily correspond to a physically independent entity. That is, these functional entities can be implemented in software form, or implemented in one or more hardware modules or integrated circuits, or implemented in different networks and/or processor devices and/or microcontroller devices.
[0083] The term "and/or" includes all combinations of any one or more of the items listed in the associated list.
[0084] Figure 1 is a schematic diagram of a drug evaluation method provided in an embodiment of the present manual, which includes:
[0085] S1: Obtaining sample information of a patient, wherein the sample information includes disease information and genetic testing results, and preprocessing the genetic testing results, wherein the genetic testing results include gene loci and genotypes, and the gene loci correspond one-to-one to the genotypes.
[0086] S2 identifies and reads multiple first gene loci and their corresponding first genotypes based on the disease information, and obtains auxiliary interpretation genes and type interpretation results, where the type interpretation results correspond one-to-one with the auxiliary interpretation genes.
[0087] S3 matches the treatment drugs based on the disease information, identifies the first auxiliary interpretation gene and/or the second gene locus associated with the treatment drugs, searches for the corresponding first type

© 2023 Kang Z et al. *JAMA Network Open*.

interpretation result for the first auxiliary interpretation gene, and identifies the second genotype corresponding to the second gene locus.
[0088] S4 interprets the first type interpretation result and/or the second genotype to obtain the drug interpretation result for the treatment drugs.
[0089] S5 generates a personalized medication evaluation plan based on all the drug interpretation results to provide reference and assist decision-making.
[0090] There is a mutual interaction between drugs and genes, mainly reflected in three aspects: 1. drug metabolism, which mainly affects the blood drug concentration and the resulting changes in drug intake; 2. drug response, which mainly affects the drug's pharmacokinetic efficiency and response efficiency, leading to changes in clinical efficacy; 3. drug adverse reactions, which mainly manifest as changes in the risk of drug-induced adverse reactions.
[0091] In most cases, doctors prescribe medications directly based on the patient's condition and instruct the patient to take the relevant treatment drugs accordingly. However, due to the patient's genetic mutations or coexisting illnesses, the effectiveness of the prescribed drugs may be suboptimal. In this case, the patient may need to try different drugs or dosages multiple times, which undoubtedly prolongs the treatment time. This application is based on the patient's disease information and genetic testing results. Through a two-step process of auxiliary gene reading and then reading genes or gene loci related to the treatment drugs, it generates a personalized clinical prescription and medication evaluation plan for the specific disease information, which doctors can refer to when prescribing medication. This helps in selecting the appropriate drugs for the patient among multiple treatment drugs, which improves patient compliance and reduces treatment costs.
[0092] S1 obtains sample information from patients, including disease information and genetic testing results. The genetic testing results are preprocessed, including genetic loci and genotypes, which are one-to-one correspondence.
[0093] Figure 2 is a schematic diagram of a medication evaluation method for preprocessing genetic testing results provided in an embodiment of this manual. The patient's sample information includes disease information and genetic testing results, and the disease information is based on the clinical diagnosis of the doctor. The genetic testing results are data obtained by the patient during genetic testing and include genetic loci and genotypes, with at least the first and second genetic loci included in the test results.
[0094] Since the genetic testing results directly affect the interpretation results in the later stage, after extracting the genetic testing results, quality control processing must be performed to ensure the integrity and compliance of the genetic loci and genotype data. Each genetic locus must conform to the preset valid data before downstream analysis and interpretation can be

© 2023 Kang Z et al. *JAMA Network Open*.

performed, thereby improving the accuracy of personalized medication evaluation plan.
[0095] In an embodiment of this manual, when preprocessing the genetic testing results, formatting and compliance checks are performed. First, the genetic testing results are formatted multiple times to output multiple formatting entries, which can be presented in the form of tables or text with gene loci and genotypes separated by special symbols.
[0096] Next, quality control is performed on the genetic testing results to determine whether the formatting entries are compliant. Compliance of the formatting entries means that the genotype corresponding to the genetic locus conforms to the preset reference genotype, the content of multiple formatting entries is completely consistent, and the genetic loci in a randomly selected formatting entry are correct.
[0097] Specifically, it is determined whether there are any missing genotypes corresponding to the genetic loci. If there are, it will be returned for manual verification. Then, the content of multiple formatting entries is compared to determine if they are completely consistent. If the content of some formatting entries is inconsistent with that of others, it will be returned for manual verification. If the content of multiple formatting entries is completely consistent, a formatting entry is randomly selected, and the genetic database is searched for the same genetic locus based on the genetic locus in the formatting entry. If there is a match, the information of the genetic locus is verified to be correct. After returning for manual verification, the problem is confirmed and corrected, and the patient's sample information is obtained again for further preprocessing. Of course, in another embodiment of this manual, the problem in the formatting entry can be directly modified after manual verification to meet the conditions of compliance of the formatting entry mentioned above.
[0098] If the genetic loci and genotype data of the formatting entries meet the conditions of compliance of the formatting entries mentioned above, the formatting entry is output as a genetic testing result entry and stored. The genetic testing result entry includes the patient's sample number, the genetic loci, and the genotypes corresponding to the genetic loci. The genetic testing result entry is stored in a database that has passed national information security level protection level 3 certification to avoid data leakage. In an embodiment of this manual, quality control can also be added to each process to increase the accuracy of the output results.
[0099] In an embodiment of this manual, the genetic testing result entry includes three columns: the patient's sample number column, the genetic locus name column, and the genotype data column (as shown in Table 1).
[0100]

Sample ID	Locus	Genotype
001	ACF rs4291	TT
001	ACE rs4646994	DD
001	ADRB1 rs1801253	CC

© 2023 Kang Z et al. *JAMA Network Open*.

001	AGTR1 rs5186	AA
001	BCRP rs2231142	GT
001	CYP2C19 rs4244285	GG
001	CYP2C9 rs1057910	AA
001	CYP2D6 rs28371725	CC
001	CYP2D6 rs1135840	GG
001	CYP2D6 rs1065852	AG
001	CYP2D6 rs1058164	CG
001	CYP3A4 rs2740574	TT
001	CYP3A5 rs776746	CT
001	GNB3 rs5443	CT
001	LDLR rs688	CC
001	NEDD4L rS4149601	GG
001	ORMI rs17650	AG
001	SLCO1B1 rS4149056	CT
001	SLCO1B1 rs2306283	GG
001	YEATS4 rs7297610	TT

[0101] (Table 1)

[0102] S2 identifies and reads multiple first gene loci and their corresponding first genotypes based on disease information, and obtains auxiliary interpretation genes and type interpretation results. The type interpretation results correspond one-to-one with the auxiliary interpretation genes.

[0103] Gene loci are unique positions on chromosomes. There are many gene loci, but there are fewer chromosomes, so there are many genes on a chromosome, arranged in a single line on the chromosome.

[0104] As the later-generated personalized medication evaluation plan serves as one of the auxiliary decision-making bases for clinical prescriptions, it needs to meet the requirements of authenticity, accuracy, and effectiveness for clinical applications. The personalized medication evaluation plan is mainly generated by comprehensively considering three aspects: drug metabolism, drug response, and adverse drug reactions. Moreover, since drug metabolism, drug response, and adverse drug reactions involve many genes, and the personalized medication evaluation plan contains a variety of treatment drugs, the genes that need to be read for different treatment drugs may overlap. The interpretation result of the auxiliary interpretation gene is determined by the results of multiple gene loci, and based on the characteristics of these genes, their interpretation has logical complexity. Therefore, to improve the reading efficiency of genes related to drug treatment, the auxiliary interpretation genes are read first.

[0105] In an embodiment of this manual, an auxiliary gene determination model is created. Medical literature and clinical trial results that have been publicly available are collected and evaluated regularly, and the literature or results with high clinical relevance and able to meet clinical needs are added to the training set for training to establish the association between the disease information and the auxiliary interpretation genes. Based on the disease

information, the auxiliary interpretation genes related to the disease information are determined through the auxiliary gene determination model.

[0106] Based on the auxiliary interpretation genes, search the first gene locus corresponding to the auxiliary interpretation gene in the genetic testing result entry, and the first gene locus includes the name of the auxiliary interpretation gene. For example, for the auxiliary interpretation gene CYP2D6, based on CYP2D6, multiple first gene loci corresponding to it can be found in Table 1, including CYP2D6 rs28371725, CYP2D6 rs1135840, CYP2D6 rs1065852, and CYP2D6 rs1058164.

[0107] Based on the first gene locus, search for the corresponding first genotype in the genetic testing result entry, and read it to obtain the type interpretation result corresponding to the auxiliary interpretation gene.

[0108] Specifically, create an auxiliary interpretation model and train it based on regularly collected medical literature and training sets established according to requirements. Then, based on the first genotype data of multiple first gene loci corresponding to the same auxiliary interpretation gene, the type interpretation result corresponding to the auxiliary interpretation gene can be obtained. The association between the first genotype data of multiple first gene loci corresponding to the same auxiliary interpretation gene and the type interpretation result is established.

[0109] In an embodiment of this manual, combining Table 1 and Table 2, if the disease of patient 001 is hypertension (disease information of patient 001), the auxiliary interpretation gene corresponding to hypertension is determined based on the auxiliary gene determination mode. Based on the auxiliary interpretation gene, the first gene locus corresponding to the genetic detection result entry is searched, the first genotype corresponding to the first gene locus is identified, and based on the first gene locus and first genotype, the type interpretation result is obtained through the auxiliary interpretation model (as shown in Table 2). Among them, the CYP2D6 gene contains four first gene loci, CYP2D6 rs28371725, CYP2D6 rs1135840, CYP2D6 rs1065852, and CYP2D6 rs1058164. According to the genotype combinations CC, GG, AG, and CG, the corresponding type interpretation result is intermediate metabolizer. CYP2C19 contains the CYP2C19 rS4244285 locus, and the type interpretation result corresponding to the genotype GG is extensive metabolizer. CYP2C9 contains the CYP2C9 rS1057910 locus, and the type interpretation result corresponding to the genotype AA is extensive metabolizer. CYP3A4 contains the CYP3A4 rS2740574 locus, and the type interpretation result corresponding to the genotype TT is extensive metabolizer. CYP3A5 contains the CYP3A5 rS776746 locus, and the type interpretation result corresponding to the genotype CT is extensive metabolizer.

[0110]

Auxiliary interpretation gene	First gene locus	First genotype	Type interpretation results

© 2023 Kang Z et al. *JAMA Network Open*.

CYP2D6	CYP2D6 rs28371725 CYP2D6 rs1135840 CYP2D6 rs1065852 CYP2D6 rs1058164	CC GG AG CG	Intermediate metabolizer
CYP2C19	CYP2C19 rs4244285	GG	Extensive metabolizer
CYP2C9	CYP2C9 rs1057910	AA	Extensive metabolizer
CYP3A4	CYP3A4 rs2740574	TT	Extensive metabolizer
CYP3A5	CYP3A5 rs776746	CT	Intermediate metabolizer

[0111] (Table 2)

[0112] In one embodiment of this manual, further processing of the data in Table 2 is performed to generate auxiliary interpretation result entries in the form of Table 3, based on the patient's sample number, gene name, and genotype interpretation results.

[0113]

Sample number	Gene	Interpretation results
001	CYP2D6	Intermediate metabolizer
001	CYP2C19	Extensive metabolizer

[0114]

001	CYP2C9	Extensive metabolizer
001	CYP3A4	Extensive metabolizer
001	CYP3A5	Intermediate metabolizer

[0115] (Table 3)

[0116] Identify the first auxiliary interpretation gene and/or the second gene locus associated with the treatment drug based on the disease information matching the treatment drug, and search for the first type interpretation result corresponding to the first auxiliary interpretation gene based on the first auxiliary interpretation gene; Identify the corresponding second genotype based on the second gene locus;

[0117] Create a drug-gene determination model, regularly collect publicly available medical literature that meets the requirements and establish a training set for training, summarize the treatment drugs related to the disease, and determine the first auxiliary interpretation gene and/or the second gene locus associated with the treatment drug. Establish the association between disease information and treatment drugs, establish the association between treatment drugs and the first auxiliary interpretation gene and/or the second gene locus, so as to match the first auxiliary interpretation gene and/or the second gene locus associated with the treatment drug through the drug gene

© 2023 Kang Z et al. *JAMA Network Open*.

determination model after obtaining the disease information. The first auxiliary interpretation gene and/or the second gene locus are drug interaction genes related to drug metabolism, response, adverse reactions, etc.
[0118] Obtain the first auxiliary interpretation gene and the second gene locus associated with the treatment drug based on the drug gene determination model. Based on the second gene locus in the gene detection result entry, search for the corresponding second genotype, and based on the first auxiliary interpretation gene in the auxiliary interpretation result entry, search for the corresponding auxiliary interpretation result for use in the second step interpretation, and then provide personalized medication advice.
[0119] The auxiliary interpretation gene is directly related to the drug. First, the drugs involved in the disease are determined through disease information, and then the required auxiliary interpretation gene is determined through the involved drugs. That is, an association of "disease-drug-auxiliary interpretation gene" is generated. There are partial overlaps, completely different, completely identical, etc. among the auxiliary interpretation genes of some drugs. Therefore, for a disease, the auxiliary interpretation genes associated with it will be used at least once in the drugs associated with the auxiliary interpretation genes, but not necessarily for a specific drug. Reading the auxiliary interpretation genes first and then the genes or gene loci related to the treatment drug is beneficial to improve the interpretation speed and reduce the interpretation cost.
[0120] Specifically, in some treatment drugs, the auxiliary interpretation gene does not affect the metabolism, response, or adverse reactions of the treatment drug. Therefore, for the interpretation of the gene locus associated with the treatment drug, the interpretation of the first auxiliary interpretation gene is not involved.
[0121] In some treatment drugs, the second gene locus does not affect the metabolism, response, or adverse reactions of the treatment drug, and only the interpretation of the first auxiliary interpretation gene is required.
[0122] In some treatment drugs, the first auxiliary interpretation gene and the second gene locus affect the metabolism, response, or adverse reactions of the treatment drug. In this case, in addition to interpreting the first auxiliary interpretation gene, the second gene locus also needs to be interpreted.
[0123] In an embodiment of this manual, olmesartan and propranolol, two treatment drugs for hypertension, are taken as examples.
[0124] As shown in Table 4, Olmesartan was tested for the second gene loci, SLCO1B1 rs2306283 and SLCO1B1 rS4149056. Based on the genetic test result entries in Table 1, the second genotypes corresponding to the second gene loci were retrieved. The second genotype of SLC01B1 rS2306283 locus was GG, and the second genotype of SLC01B1 rs4149056 locus was CT.
[0125]

Treatment drugs	Second gene locus	Second genotypes
Olmesartan	SLCO1B1	GG

© 2023 Kang Z et al. *JAMA Network Open*.

	SLCO1B1	CT

[0126] (Table 4)

[0127] As shown in Table 5, the first auxiliary interpretation gene for detecting CYP2D6 in propranolol testing is obtained from the auxiliary interpretation results of CYP2D6 in the auxiliary interpretation result entries.

[0128]

Treatment drug	First auxiliary interpretation gene	First auxiliary interpretation result
Propranolol	CYP2D6	Intermediate metabolizer

[0129] (Table 5)

[0130] Obtain the drug interpretation result of the treatment drug based on the first type interpretation result and/or the second genotype of S4 reading.

[0131] In an embodiment of this manual, a comprehensive characteristic value is first generated based on the first auxiliary interpretation gene and/or the second gene locus, and the comprehensive characteristic value = index_1 * SNP_1 + index_2 * SNP_2 + ... + index_N * SNP_N. Here, the index value is the basic characteristic value of the second gene locus or the first auxiliary interpretation gene obtained based on the characteristic value database, and the basic characteristic value is determined based on the importance of the second gene locus or the first auxiliary interpretation gene to the use of the treatment drug. The higher the importance of the second gene locus or the first auxiliary interpretation gene to the use of the treatment drug, the greater the absolute value of the corresponding index. The SNP value is the weight value of the second gene locus or the first auxiliary interpretation gene obtained based on the characteristic value database, and the weight value is proportional to the strength of the mutation, that is, the stronger the mutation of the second gene locus or the first auxiliary interpretation gene, the greater the absolute value of the weight.

[0132] Then, based on the comprehensive characteristic value, the comprehensive medication recommendations and recommendation levels for the treatment drug are obtained. The comprehensive medication recommendations corresponding to the comprehensive characteristic value are obtained from the characteristic value database, and the recommendation levels corresponding to the comprehensive characteristic value are obtained from the characteristic value database.

[0133] Based on the interval where the comprehensive characteristic value is located, the recommended level of the treatment drug is determined as prioritized recommended, generally recommended, or cautiously recommended. The recommended levels include prioritized recommended, generally recommended, and cautiously recommended, and the three recommendation rates decrease in turn. The interval where the comprehensive characteristic values of multiple treatment drugs correspond to the recommended levels is separately set.

© 2023 Kang Z et al. *JAMA Network Open*.

[0134] In an embodiment of this manual, when the comprehensive characteristic value of the preset treatment drug M is in the interval (a, b], the corresponding recommended level is prioritized recommended; when the comprehensive characteristic value of the treatment drug M is in the interval (b, c], the corresponding recommended level is generally recommended; when the comprehensive characteristic value of the treatment drug M is in the interval (c, d], the corresponding recommended level is cautiously recommended ($a<b<c<d$).
[0135] In an embodiment of this manual, the interval where the comprehensive characteristic values of each treatment drug correspond to the recommended levels is different. That is, when the comprehensive characteristic value of the preset treatment drug N is in the interval (a, e], the corresponding recommended level is prioritized recommended; when the comprehensive characteristic value of the treatment drug N is in the interval (e, f], the corresponding recommended level is generally recommended; when the comprehensive characteristic value of the treatment drug N is in the interval (f, g], the corresponding recommended level is cautiously recommended ($a<e<f<g$).
[0136] The recommended level corresponding to the comprehensive characteristic value varies depending on different treatment drugs, which means that the same comprehensive characteristic value of different treatment drugs may have the same or different corresponding recommended levels. For example, if the comprehensive characteristic values of the treatment drug M and the treatment drug N are both h, and assuming $b < h < c$, the recommended level of the treatment drug M is generally recommended, while the recommended level of the treatment drug N is prioritized. Although their comprehensive characteristic values are the same, their recommended levels are different.
[0137] In one embodiment of this manual, the three recommended levels of prioritized, generally, and cautiously recommended can be respectively marked as 1, 2, 3; or marked as one, two, three; or marked as 75%, 50%, 25%; or marked as various forms such as "priority use according to the instruction manual", "cautiously use", "cautiously use with frequent monitoring" to distinguish between the recommended levels.
[0138] The drug interpretation results include the comprehensive feature value, the comprehensive medication recommendation, and the recommendation level. The comprehensive feature value is obtained based on the basic feature value and weight value through the comprehensive feature value model.
[0139] In one embodiment of this manual, considering the nonlinear non-additive relationship between gene loci, the comprehensive feature value model includes non-linear processing of the basic feature value and weight value. In another embodiment of this manual, since the formula calculation itself can be used as a means of linearization, the comprehensive feature

© 2023 Kang Z et al. *JAMA Network Open*.

value model also includes linear processing of the basic feature value and weight value. Specifically, linear weighting is performed on the basic feature value and weight value. In one embodiment of this manual, based on the drug interpretation results for each treatment drug, the medication recommendation items for the patient are generated.

[0140] In one embodiment of this manual, if the first auxiliary interpretation gene and the second gene locus are interpreted, then:

[0141] Obtain the basic feature value and weight value of the first auxiliary interpretation gene based on the first auxiliary interpretation gene and the first type of interpretation result obtained from the first auxiliary interpretation gene;

[0142] Obtain the basic feature value and weight value of the second gene locus based on the second gene locus and the second genotype;

[0143] Based on the basic feature value and weight value of each first auxiliary interpretation gene and each second gene locus of the treatment drug, generate the comprehensive feature value of the treatment drug through the comprehensive feature value model;

[0144] Obtain the comprehensive medication recommendation and recommendation level based on the comprehensive feature value;

[01451 The drug interpretation results include the comprehensive feature value, the comprehensive medication recommendation, and the recommendation level.

[0146] In one embodiment of this manual, if only the second gene locus is interpreted, then:

[0147] Obtain the basic feature value and weight value of the second gene locus based on the second gene locus and the second genotype;

[0148] Based on the basic feature value and weight value of each second gene locus of the treatment drug, generate the comprehensive feature value of the treatment drug through the comprehensive feature value model;

[0149] Obtain the comprehensive medication recommendation and recommendation level based on the comprehensive feature value;

[0150] The drug interpretation results include the comprehensive feature value, the comprehensive medication recommendation, and the recommendation level.

[0151] In one embodiment of this manual, as shown in Table 4, for the treatment drug olmesartan, the comprehensive feature value needs to be obtained based on the second genotype of SLC01B1 rs2306283 and SLCO1B1 rs4149056. Based on the feature value database, the index value (basic feature value) of the SLC01B1 rs2306283 is 10, and the index value (basic feature value) of the SLCO1B1 rs4149056 locus is 12. The SNP values (weight values) of the SLC01B1 rs2306283 genotypes GG, AG, and AA are 1, 1.2, and 2, respectively. The SNP values (weight values) of the SLC01B1 rs4149056 genotypes TT, CT, and CC are 1, 2.5, and 3, respectively.

© 2023 Kang Z et al. *JAMA Network Open*.

[0152] Since the second genotype of patient 001's SLC01B1 rs2306283 is GG, its SNP value (weight value) is 1; since the second genotype of patient 001's SLCO1B1 rs4149056 is CT, its SNP value (weight value) is 2.5;
[0153] For patient 001, based on the basic feature values and weight values, a linear weighting is performed, and the comprehensive feature value of olmesartan is 10 * 1+12 * 2.5 = 40.
[0154] Based on the comprehensive feature value, the comprehensive medication recommendation for the patient is obtained through the feature value database: if the patient take olmesartan, the initial dose should be appropriately reduced, followed by observation of clinical treatment effects and dose adjustment. Based on the comprehensive feature value obtained from the feature value database, olmesartan is recommended for cautiously use for patient 001.
[0155] In one embodiment of this manual, if only the first auxiliary genotyping is read;
[0156] Based on the first auxiliary genotyping and the first type of genotyping result, obtain the basic feature value and weight value of the first auxiliary genotyping.
[0157] The basic feature values and weight values of each first auxiliary interpretation gene based on the treatment drug are used to generate the comprehensive feature value of the treatment drug through a comprehensive feature value model.
[01581] The comprehensive medication recommendations and recommendation levels are obtained based on the comprehensive feature value.
[0159] The drug interpretation result includes the comprehensive feature value, the comprehensive medication recommendations, and the recommendation level.
[0160] In an embodiment of the present manual, for the treatment drug propranolol, a comprehensive feature value is obtained based on the first type interpretation result of CYP2D6, as shown in Table 5. Based on the feature value database, the index value (basic feature value) of the CYP2D6 gene is 10, and the typing SNP values (weight values) for CYP2D6 ultrarapid metabolizers, extensive metabolizers, intermediate metabolizers, and poor metabolizers are 2, 1, 0.8, and 0.2, respectively.
[0161] Since the first type interpretation result of patient 001's CYP2D6 gene corresponds to the intermediate metabolizer type, its SNP value (weight value) is 0.8.
[0162] For patient 001, based on the basic feature value and weight value, the comprehensive feature value of propranolol is 10 * 0.8 = 8.
[0163] Based on the comprehensive feature value, the comprehensive medication recommendation for the patient is obtained through the feature value database: if the patient take propranolol, the initial dose should be appropriately reduced. Clinical treatment effects should be observed and dose

© 2023 Kang Z et al. *JAMA Network Open*.

adjustments should be made. Based on the comprehensive feature value, the recommended level for propranolol for patient 001 is cautious use.
[0164] The medication advice for patient 001 is shown in Table 6:
[0165]

Treatment drugs	Feature value	Comprehensive medication recommendations	Recommendation level
Olmesartan	40	If the patient takes olmesartan, the initial dose should be appropriately reduced. Clinical treatment effects should be observed and dose adjustments should be made.	Cautiously use
Propranolol	8	If the patient takes propranolol, the initial dose should be appropriately reduced. Clinical treatment effects should be observed and dose adjustments should be made.	Cautiously use

[0166] (Table 6)
[0167] S5 combines the drug interpretation results of all the treatment drugs to generate a personalized medication evaluation plan for reference and decision-making assistance.
[0168] In an embodiment of the present manual, all the drug interpretation results of the treatment drugs are summarized, and the treatment drugs are classified based on the recommended level to generate a personalized medication evaluation plan, which provides guidance and suggestions for adjusting the type and dosage of medication for the patient to reduce adverse reactions caused by medication.
[0169] In another embodiment of the present manual, patient 002's disease is hyperlipidemia (patient 002's disease information), and the genetic testing result entries based on the genetic testing results are shown in Table 7.
[0170]

Sample number	Gene locus	Genotypes
002	CYP3A5 rs776746	CT
002	ABCB1 rs1045642	GG
002	ABCB1 rs2032582	AC
002	ABCG2 rs2231142	GT
002	APOE rs429358	TT
002	APOE rs7412	CC
002	CETP rs5882	AG
002	CYP2C9 rs1057910	AA
002	CYP3A4 rs35599367	GG
002	CYP3A4 rs2740574	TT
002	CYP3A4 rs2242480	CT
002	HMGCR rs17244841	AA

© 2023 Kang Z et al. *JAMA Network Open*.

002	LEPR rs1137101	GG
002	LIPC rs1800588	CC
002	MTHFR rs1801133	AG
002	SLCO1B1 rs4149056	TT
002	SLCO1B1 rs2306283	GG
002	SLCO1B1 rs4149015	GG

[0170] (Table 7)

[0172] Based on the technical solutions described in this manual, the format of personalized medication evaluation scheme can be shown in

[0173] Table 8:

[0174]

Priority use according to the instructions	Use with caution	Use with caution and monitor frequently
Lovastatin	Simvastatin<br>Fluvastatin<br>Atorvastatin<br>Rosuvastatin	Pravastatin

[0175]

	Pitavastatin	

[0176] (Table 8)

[0177] To assist doctors in determining personalized medication plans for patients, based on the first auxiliary interpretation gene and/or the second gene locus, gene interpretation results can also be obtained in an embodiment of this manual.

[0178] Specifically, a gene interpretation model is created, and publicly available medical literature and clinical trial results are periodically collected and added to the training set for training purposes. The corresponding relationships between various second gene types corresponding to the second gene locus and gene interpretation results are established, as well as the corresponding relationships between the first type of interpretation results corresponding to the first auxiliary interpretation gene and gene interpretation results.

[0179] Based on the first type of interpretation results corresponding to the first auxiliary interpretation gene, the first gene interpretation result is obtained through the gene interpretation model. Based on the second gene type corresponding to the second gene locus, the second gene interpretation result is obtained through the gene interpretation model.

[0180] If the drug interaction genes of the treatment drug include the second gene locus and the first auxiliary interpretation gene, all first interpretation results and all second interpretation results of the treatment drug are summarized.

[0181] If the drug interaction genes of the treatment drug include only the first auxiliary interpretation gene, summarize all the first interpretation results of the treatment drug.

© 2023 Kang Z et al. *JAMA Network Open.*

[0182] If the drug interaction genes of the treatment drug only include the second gene locus, summarize all the second interpretation results of the treatment drug.
[0183] In an embodiment of this manual, combined with Table 4, for patient 001, the second gene locus of olmesartan includes SLCO1B1 rs2306283 and SLCO1B1 rs4149056.
[0184] Among them, the genotype of patient 001's SLCO1B1 rs2306283 is GG, and the corresponding gene interpretation result obtained by the gene interpretation model is “the SLCO1B1-I genotype of patient 001 is: mutant homozygous (GG), the clearance rate of olmesartan is normal.”
[0185] The genotype of patient 001's SLCO1B1 rs4149056 is CT, and the corresponding gene interpretation result obtained by the gene interpretation model is "the SLCO1B1-II genotype of patient 001 is: mutant heterozygous (CT), the clearance rate of olmesartan decreases and the blood drug concentration increases."
[0186] In another embodiment of this manual, combined with Table 5, for patient 001, the first auxiliary interpretation gene of pindolol includes CYP2D6. Among them, the type interpretation results of patient 001's CYP2D6 gene is GG, and the corresponding gene interpretation result obtained by the gene interpretation model is “the CYP2D6 genotype of patient 001 is: intermediate metabolizer, and the metabolism rate of pindolol partially decreases.”
[0187] Summarize all the first interpretation results and/or all the second interpretation results of the treatment drugs to generate gene interpretation entries. The gene interpretation entry includes the name of the treatment drug, the second gene locus, the second genotype, and the gene interpretation result; or the gene interpretation entry includes the name of the treatment drug, the first auxiliary interpretation gene, the first type interpretation result, and the gene interpretation result; or the gene interpretation entry includes the name of the treatment drug, the second gene locus, the first auxiliary interpretation gene, the second genotype, the first type interpretation result, and the gene interpretation result.
[0188] In another embodiment of this manual, combined with Tables 4 and 5, the gene interpretation result entry of patient 001 obtained is shown in Table 9:
[0189]

Drug	Gene locus	Genotype	Gene interpretation result
Olmesartan	SLCO1B1 rs2306283	CG	The SLCO1B1-I genotype of patient 001 is: mutant homozygous (GG), and the clearance rate of olmesartan is normal.
	SLCO1B1 rs4149056	CT	The SLCO1B1-II genotype of patient 001 is: mutant heterozygous (CT), the clearance rate of olmesartan

© 2023 Kang Z et al. *JAMA Network Open*.

			decreases and the blood drug concentration increases.
Pindolol	CYP2D6	Intermediate metabolizer	The CYP2D6 genotype of patient 001 is: intermediate metabolizer, and the metabolism rate of pindolol partially decreases.

[0190] (Table 9)

[0 191] In another embodiment of this manual, the gene interpretation results are summarized into personalized medication evaluation plan in conjunction with Table 8. The corresponding treatment drugs are highlighted in the upper right corner with numbers, and specific treatment drugs for patient 002 are described in terms of metabolism, response, and adverse reactions. Among them, 1 represents normal drug metabolism; 2 represents decreased drug metabolism; 3 represents increased drug metabolism; 4 represents normal drug efficacy; 5 represents decreased drug efficacy; 6 represents increased drug efficacy; 7 represents normal drug toxicity; 8 represents decreased drug toxicity; and 9 represents increased drug toxicity. As shown in Table 10:

[0192]

Priority use according to the instructions	Use with caution	Use with caution and monitor frequently
Lovastatin [1, 6]	Simvastatin [1, 5], Fluvastatin [2, 5], Atorvastatin [2, 6, 7],	Pravastatin [1, 4, 8]

[0193]

	Rosuvastatin [2, 6], Pitavastatin [2]	

[0194] Note: 1. Normal drug metabolism; 2. Decreased drug metabolism; 3. Increased drug metabolism; 4. Normal drug efficacy; 5. Decreased drug efficacy; 6. Increased drug efficacy; 7. Normal drug toxicity; 8. Decreased drug toxicity; and 9. Increased drug toxicity.

[0195] (Table 10)

[0196] In one embodiment of this manual, the personalized medication evaluation plan is output in the form of a text report. Of course, the text report can also include one or more of the genetic testing result items, auxiliary interpretation result items, and gene interpretation items.

[0197] In this manual, the main characteristics of the genes/gene loci that need to be interpreted are that the activity changes are greatly influenced by genetic mutations, and the final effect on drugs will also have a significant impact. There are many related mutations, including coding and non-coding regions of genes. The influence on gene activity may be positive or negative, and the gene activity is ultimately determined by the comprehensive results of multiple mutations. Due to the different importance of mutations related to genes, weights are based on the basic characteristic values to distinguish different gene loci. Moreover, in the data collation process and interpretation process, quality control can be based on multiple output results to reduce artificial intervention, ensure the stability and accuracy of data output, and

© 2023 Kang Z et al. *JAMA Network Open*.

fully protect patient privacy. Compared with manual interpretation, the medication evaluation plan generation method in this manual reduces the demand for manual labor and training, greatly reducing labor costs.
[0198] In summary, obtaining patient disease information, extracting and storing raw genotype testing data, and improving the accuracy of later interpretation based on quality control processes; through the two-step interpretation of the first gene locus and the drug interaction gene (the second gene locus and/or the first auxiliary interpretation gene), a personalized medication evaluation plan is obtained, which shortens the interpretation time and improves the overall efficiency of interpretation. It is filled in the given report format to generate a text version of the interpretation report, which is convenient for downloading and checking, and provides assistance for doctors to prescribe medication.
[0199] Although the examples provided in this manual for patient 001 (hypertension) and patient 002 (hyperlipidemia) are extracted, organized and stored based on the genotype data of related chronic disease drugs' gene interaction loci, and personalized medication assessment schemes are obtained through secondary interpretation to avoid some medication risks for chronic disease patients, this method for generating personalized medication assessment schemes can also be applied in other non-chronic disease fields, which will not be further illustrated here.
[0200] Figure 3 shows a structural diagram of a medication evaluation system provided in an embodiment of this manual. The system includes:
[0201] Acquisition module, used for acquiring patient sample information, including disease information and genetic test results, preprocessing the genetic test results, including gene loci and genotypes, and each gene locus corresponds to one genotype.
[0202] Auxiliary interpretation module, used to identify and interpret multiple first gene loci and their corresponding first genotypes based on disease information, and obtain auxiliary interpretation genes and type interpretation results, where each type interpretation result corresponds to one auxiliary interpretation gene.
[0203] Recognition module, used to match treatment drugs based on the disease information, identify the first auxiliary interpretation gene and/or the second gene locus associated with the treatment drugs, and find the corresponding first type interpretation result based on the first auxiliary interpretation gene; and identify the second genotype corresponding to the second gene locus.
[0204] Drug interpretation module, used to interpret the first type interpretation result and/or the second genotype and obtain the drug interpretation result for the treatment drug.
[0205] Scheme generation module, used to generate a personalized medication assessment scheme by combining the drug interpretation results of multiple treatment drugs for reference and auxiliary decision-making.

© 2023 Kang Z et al. *JAMA Network Open*.

[0206] Optionally, the acquisition module includes:
[0207] Formatting sub-module, used to format the genetic test results repeatedly to generate multiple formatting entries.
[0208] Judgment sub-module, used to judge whether the formatting entries are compliant.
[0209] Output sub-module, used to output the formatting entries as genetic test result entries and store them if the formatting entries are compliant, where the genetic test result entries include the patient's sample number, gene locus, and corresponding genotype.
[0210] Optional, the auxiliary interpretation module includes:
[0211] The auxiliary gene determination sub-module is used to determine the auxiliary interpretation gene based on the disease information.
[0212] The identification sub-module is used to identify multiple first gene loci and their corresponding first genotypes based on the auxiliary interpretation gene.
[0213] The auxiliary interpretation sub-module is used to interpret all first gene loci and first genotypes of the auxiliary interpretation gene and obtain the type interpretation result corresponding to the auxiliary interpretation gene.
[0214] Optional, the drug interpretation module includes:
[0215] The first acquisition sub-module is used to obtain the basic feature values and weight values of the first auxiliary interpretation gene based on the first auxiliary interpretation gene and the first type interpretation result.
[0216] The first feature value generation sub-module is used to generate the comprehensive feature value of the treatment drug based on the basic feature values and weight values of each first auxiliary interpretation gene of the treatment drug through a comprehensive feature value model.
[0217] The first recommendation sub-module is used to obtain comprehensive medication advice and recommendation level based on the comprehensive feature value.
[0218] The drug interpretation result includes the comprehensive feature value, the comprehensive medication advice, and the recommendation level.
[0219] Optional, the drug interpretation module includes:
[0220] The second acquisition sub-module is used to obtain the basic feature values and weight values of the second gene locus based on the second gene locus and the second genotype.
[0221] The second feature value generation sub-module is used to generate the comprehensive feature value of the treatment drug based on the basic feature values and weight values of each second gene locus of the treatment drug through a comprehensive feature value model.
[0222] The second recommendation sub-module is used to obtain comprehensive medication advice and recommendation level based on the comprehensive feature value.
[0223] The drug interpretation result includes the comprehensive feature value, the comprehensive medication advice, and the recommendation level.

© 2023 Kang Z et al. *JAMA Network Open*.

[0224] Optional, the drug interpretation module includes:
[0225] The third acquisition sub-module is used to obtain the basic feature values and weight values of the first auxiliary interpretation gene based on the first auxiliary interpretation gene and the first type interpretation result.
[0226] The fourth acquisition sub-module is used to obtain the basic feature values and weight values of the second gene locus based on the second gene locus and the second genotype.
[0227] The third feature value generation sub-module is used to generate the comprehensive feature value of the treatment drug based on the basic feature values and weight values of each first auxiliary interpretation gene and each second gene locus of the treatment drug through a comprehensive feature value model.
[0228] The third recommendation sub-module is used to obtain comprehensive medication advice and recommendation level based on the comprehensive feature value.
[0229] The drug interpretation result includes the comprehensive feature value, the comprehensive medication advice, and the recommendation level.
[0230] Optional, the recommendation level of the treatment drug is determined as prioritized recommended, generally recommended, or cautiously recommended based on the interval in which the comprehensive feature value falls. The interval of the comprehensive feature value corresponding to different recommendation levels of different treatment drugs is different.
[0231] The functions of the system in the embodiment of the present invention have been described in the above method embodiment.
[0232] Optional, the drug interpretation module further comprises:
[0233] The fifth acquisition sub-module, which is used to obtain the basic feature values and weight values of the third gene locus based on the third gene locus and the third genotype.
[0234] The fourth feature value generation sub-module, which is used to generate the comprehensive feature value of the treatment drug based on the basic feature values and weight values of each of the first auxiliary interpretation genes, each of the second gene loci, and each of the third gene loci of the treatment drug through a comprehensive feature value model.
[0235] The fourth recommendation sub-module, which is used to obtain comprehensive medication recommendations and recommendation levels based on the comprehensive feature value.
[0236] The drug interpretation results include the comprehensive feature value, comprehensive medication recommendations, and recommendation levels.
[0237] The functions of the system in the embodiments of the present invention have been described in detail in the above method embodiments and will not be repeated here.

Figures

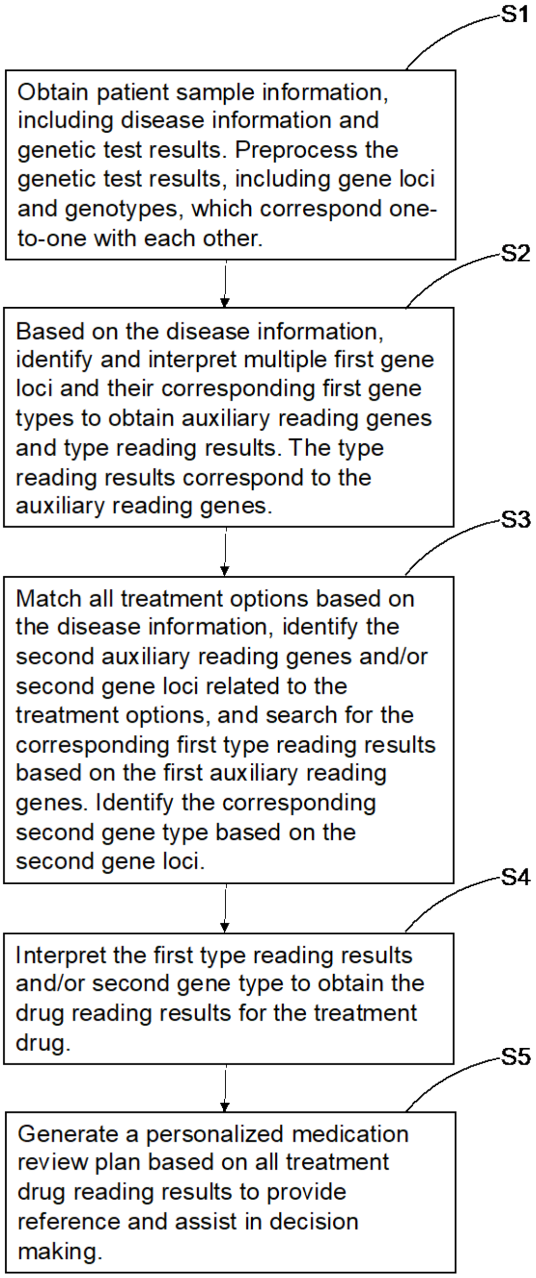

Figure 1

© 2023 Kang Z et al. *JAMA Network Open*.

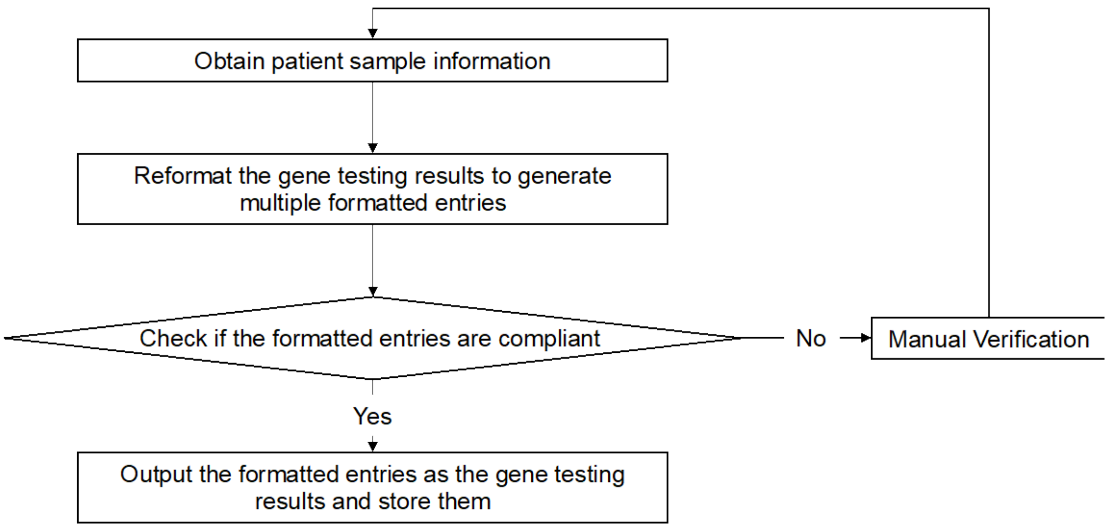

Figure 2

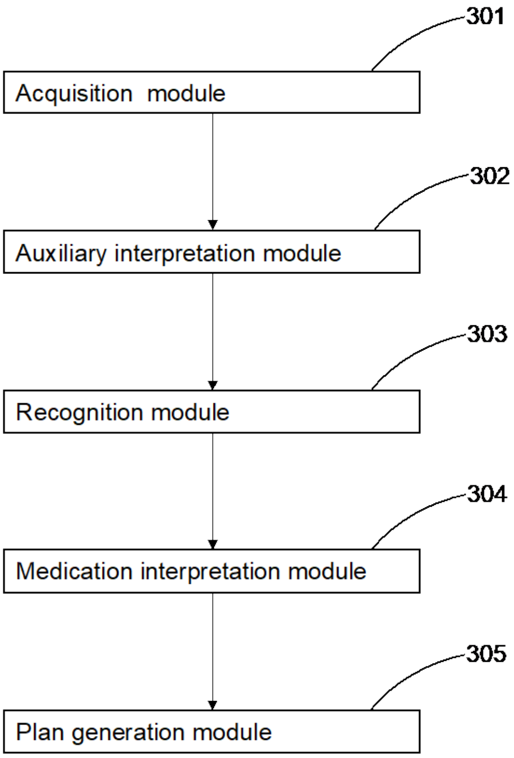

Figure 3

© 2023 Kang Z et al. *JAMA Network Open*.

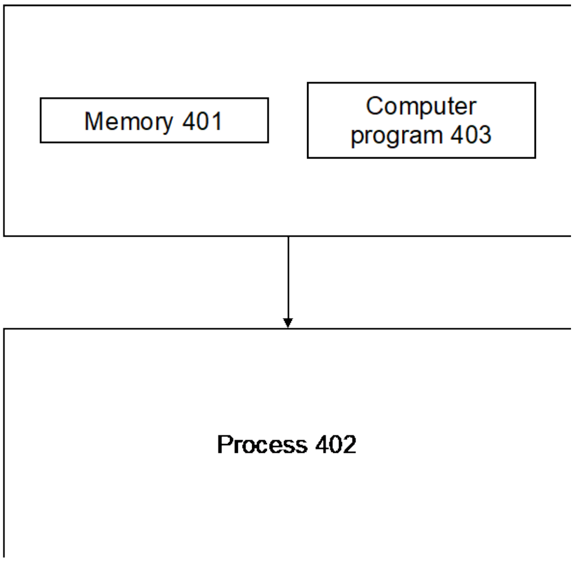

Figure 4

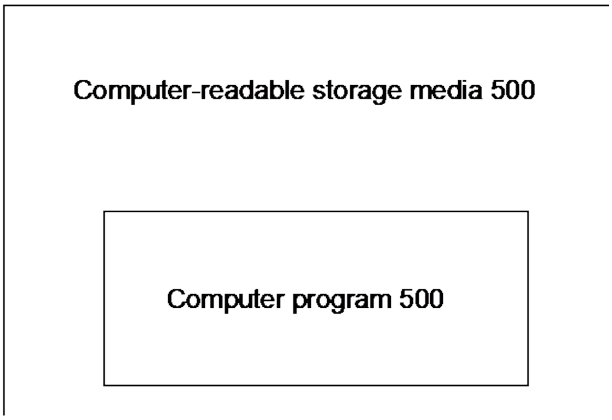

Figure 5

© 2023 Kang Z et al. *JAMA Network Open*.
