# Supplementary material for: Multigenetic Pharmacogenomics–Guided Treatment vs Treatment As Usual Among Hospitalized Men With Schizophrenia: A Randomized Clinical Trial
Source: JAMA Netw Open. 2023 Oct 6;6(10):e2335518. doi: 10.1001/jamanetworkopen.2023.35518 (PMC10559185; doi:10.1001/jamanetworkopen.2023.35518)
Supplement: Supplement 3. — Data Sharing Statement [file jamanetwopen-e2335518-s003.pdf]

Data Sharing Statement

Kang. Multigenetic Pharmacogenomics–Guided Treatment vs Treatment As Usual Among Hospitalized Men With Schizophrenia. *JAMA Netw Open*. Published October 06, 2023. doi:10.1001/jamanetworkopen.2023.35518

Data
Data available: Yes
Data types: Deidentified participant data
How to access data: The data presented in this study are available on request from the corresponding author, Prof. Weihua Yue. E-mail: dryue@bjmu.edu.cn
When available: With publication

Supporting Documents
Document types: None

Additional Information
Who can access the data: The data will be made available for to researchers whose proposed use of the data has been approved.
Types of analyses: The data will be made available for specified purposes, which include further research, replication of findings, and exploration of related research questions within the scope of the study. However, access to the data will be subject to appropriate data sharing agreements, ethical considerations, and compliance with relevant regulations and policies.
Mechanisms of data availability: The data will be made available with investigator support and after approval of a data access proposal.
